# Epigenetic changes around the pX region and spontaneous HTLV-1 transcription are CTCF-independent

**DOI:** 10.12688/wellcomeopenres.14741.2

**Published:** 2018-12-11

**Authors:** Michi Miura, Paola Miyazato, Yorifumi Satou, Yuetsu Tanaka, Charles R.M. Bangham

**Affiliations:** 1 Division of Infectious Diseases, Department of Medicine, Imperial College London, London, W2 1PG, UK; 2 Center for AIDS Research, Kumamoto University, Kumamoto, 860-0811, Japan; 3 Department of Immunology, University of the Ryukyus, Okinawa, 903-0215, Japan

**Keywords:** Histone modifications, DNA methylation, Epigenetics, Antisense transcription, Transcription kinetics, Retrovirus, HTLV-1, CTCF, CRISPR/Cas9, Single-molecule RNA-FISH

## Abstract

**Background:** The human retrovirus HTLV-1 inserts the viral complementary DNA of 9 kb into the host genome. Both plus- and minus-strands of the provirus are transcribed, respectively from the 5′ and 3′ long terminal repeats (LTR). Plus-strand expression is rapid and intense once activated, whereas the minus-strand is transcribed at a lower, more constant level. To identify how HTLV-1 transcription is regulated, we investigated the epigenetic modifications associated with the onset of spontaneous plus-strand expression and the potential impact of the host factor CTCF.

**Methods:** Patient-derived peripheral blood mononuclear cells (PBMCs) and in vitro HTLV-1-infected T cell clones were examined. Cells were stained for the plus-strand-encoded viral protein Tax, and sorted into Tax ^+^ and Tax ^–^ populations. Chromatin immunoprecipitation and methylated DNA immunoprecipitation were performed to identify epigenetic modifications in the provirus. Bisulfite-treated DNA fragments from the HTLV-1 LTRs were sequenced. Single-molecule RNA-FISH was performed, targeting HTLV-1 transcripts, for the estimation of transcription kinetics. The CRISPR/Cas9 technique was applied to alter the CTCF-binding site in the provirus, to test the impact of CTCF on the epigenetic modifications.

**Results:** Changes in the histone modifications H3K4me3, H3K9Ac and H3K27Ac were strongly correlated with plus-strand expression. DNA in the body of the provirus was largely methylated except for the pX and 3′ LTR regions, regardless of Tax expression. The plus-strand promoter was hypomethylated when Tax was expressed. Removal of CTCF had no discernible impact on the viral transcription or epigenetic modifications.

**Conclusions:** The histone modifications H3K4me3, H3K9Ac and H3K27Ac are highly dynamic in the HTLV-1 provirus: they show rapid change with the onset of Tax expression, and are reversible. The HTLV-1 provirus has an intrinsic pattern of epigenetic modifications that is independent of both the provirus insertion site and the chromatin architectural protein CTCF which binds to the HTLV-1 provirus.

## Introduction

Human T cell leukemia virus type 1 (HTLV-1) was the first pathogenic exogenous retrovirus identified in humans. The main routes of infection are breast feeding, sexual contact and blood transfusion, each of which transmits cells carrying HTLV-1 and capable of infecting other cells in a new host. The majority of infected individuals remain asymptomatic throughout life. However some 5% develop adult T cell leukemia (ATL), and up to another 5% develop HTLV-1-associated myelopathy/tropical spastic paraparesis (HAM/TSP) ^1, 2^.

HTLV-1 reverse-transcribes its 9 kb genomic RNA into complementary double-stranded DNA which is then inserted into the host cellular DNA upon infection. Thereafter the virus remains as a chromatinized provirus and is replicated as a part of the host genome. The virus mainly resides in CD4 ^+^ T cells. Each infected cell carries a single copy of the HTLV-1 provirus in a given location in the host genome ^3, 4^.

**Figure 1.**
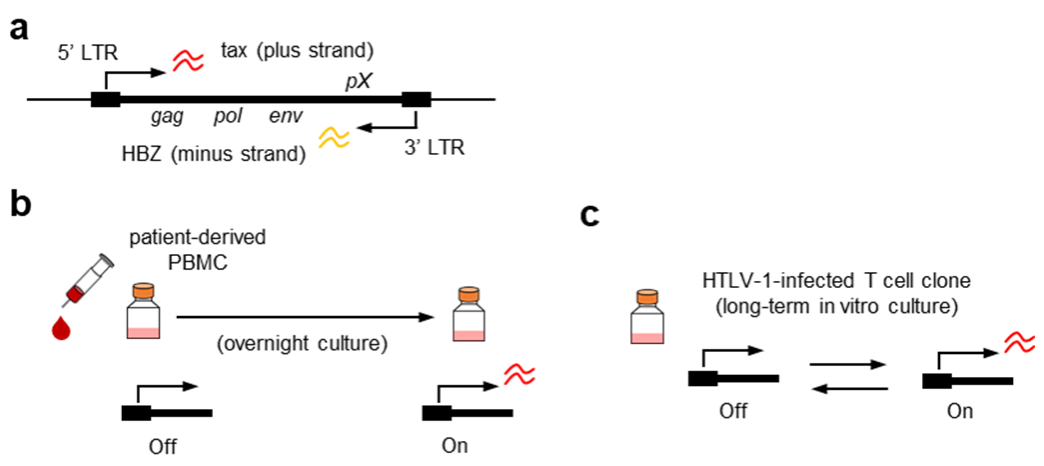
HTLV-1 transcription in two distinct models. **a**) Schematic diagram of HTLV-1 provirus inserted in the host genome. The HTLV-1 provirus has two identical LTRs, one at each end of the provirus. As well as genes encoding the canonical retroviral structural components Gag, Pol and Env, the provirus contains a group of regulatory genes in the pX region on the plus-strand. The plus-strand transcripts, represented by *tax*, are coloured in red, and the minus-strand transcript *HBZ* in yellow. ( **b**) In PBMCs freshly isolated from HTLV-1 carriers, HTLV-1 reactivates and expresses the plus-strand transcripts within a few hours of culture; but these transcripts remain transcriptionally silent for most of the time *in vivo*. ( **c**) In HTLV-1-infected T cell clones cultured *in vitro*, the promoter activity for plus-strand transcripts shuttles between the on and off state. The plus-strand transcripts are only produced when the promoter activity is on, yielding only a limited fraction of cells that are positive for the plus-strand transcripts at a given time.

The provirus has identical long terminal repeats (LTRs) at the 5′ and 3′ ends, each of which serves as a promoter to drive the transcription of HTLV-1 from the plus- and minus-strand, respectively ( Figure 1a) (reviewed in ref. 5). Most of the viral transcripts are from the plus strand: the transcripts yield a variety of viral proteins by alternative splicing. One of the plus-strand products is Tax, which has diverse functions in the infected cells including immortalization ^6, 7^, accelerated cell cycle progression into S phase ^8^, cell proliferation ^9^ and DNA damage ^10^. Tax also exerts a strong positive feedback on the promoter in the 5′ LTR by assembling other transcription activators (reviewed in ref. 5). HTLV-1 encodes HBZ in the minus strand from the 3′ LTR ^11^ ( Figure 1a). This transcript also contributes to viral pathogenesis ^12^.

In HTLV-1-infected individuals, *tax* is often undetectable in freshly isolated peripheral blood mononuclear cells (PBMCs), whereas *HBZ* is almost invariably detectable ^13^, revealing a sharp contrast between the plus- and minus-strand transcription. However, the evidence of constitutive reactivity of cytotoxic T cells (CTLs) against Tax *ex vivo* ^14^ indicates that CTLs are frequently exposed to the viral antigen Tax *in vivo*, suggesting that *tax* is intermittently expressed *in vivo*. The current view of HTLV-1 expression *in vivo* is that *tax* is usually silenced but undergoes intermittent expression, whereas *HBZ* is almost constantly expressed, yet at a low level ^1^.

HTLV-1 expression in fresh, patient-derived PBMCs follows a characteristic trajectory. Once PBMCs are isolated from the blood of infected individuals and put into culture, a fraction of infected cells start expressing Tax within the first few hours ^15^ ( Figure 1b), perhaps triggered by the stress experienced by the cells on removal from the circulation ^16^. Plus-strand bursts are triggered by cell stress via p38 MAPK activation and require deubiquitylation at the HTLV-1 promoter of histone H2A (Lys119), the inhibitory transcriptional mark characteristic of polycomb repressive complex 1 (PRC1) ^16^. In patient-derived HTLV-1-infected clones *in vitro*, plus-strand bursts are more frequent in cells that lack *HBZ* mRNA and in cells in G0/G1 phase ^17^, and more intense under conditions of physiological hypoxia ^18^. In contrast, the abundance of *HBZ* mRNA remains relatively stable during short-term culture *in vitro* ( Figure 1b).

In addition to examining fresh PBMCs, we investigated HTLV-1 transcription in HTLV-1-infected T cell clones established from patient-derived PBMCs ^4^. These clones behave differently from the *ex vivo* PBMCs described above. We recently examined the plus- and minus-strand expression simultaneously at the single-cell level in these clones, by single-molecule RNA-FISH ^17^. The plus strand shows a rapid and intense expression, often referred to as a burst, seemingly flanked by a period of transcriptional silence. In a clonal cell population, at any given time, a plus-strand burst is present in a fraction (5% to 30%) of cells, each cell containing hundreds of transcripts, leaving the other cells negative for the plus-strand expression ( Figure 1c). On the other hand, *HBZ* is expressed relatively constantly, again providing evidence of asymmetric expression from the plus- and minus-strand ( Figure 1c). The simplest interpretation of these observations is that the kinetics of expression has reached equilibrium state at the population level, and that each cell in these HTLV-1-infected T cell clones continually switch on and off the 5′ LTR promoter activity and so go through cycles of intermittent plus-strand expression.

We discovered that the host protein CTCF binds to the HTLV-1 provirus in the middle of the pX region ^19^. CTCF is a chromatin-binding zinc-finger protein with a wide range of functions, including transcription regulation, insulation for repressive histone modifications, and chromatin looping. We postulated that the binding of CTCF regulates the epigenetic modifications in the provirus, and hence viral transcription.

In the present study we had two aims. First, to investigate the epigenetic modifications in the HTLV-1 provirus that accompany the dynamic changes in viral transcription during short-term culture of PBMCs, and in HTLV-1-infected T cell clones *in vitro*. Second, to examine the potential impact of CTCF on the epigenetic modifications and viral transcription. We altered the CTCF-binding site in the provirus with CRISPR/Cas9 technique to remove CTCF from the provirus, identified the epigenetic modifications and assayed viral transcription.

## Methods

### Cell culture

Peripheral blood mononuclear cells (PBMCs) from patients with the HTLV-1-associated inflammatory disease HAM/TSP were separated from peripheral blood with Histopaque (Sigma, H8889), washed in PBS, frozen in fetal bovine serum containing 10% DMSO and stored in liquid nitrogen until use. Upon thawing PBMCs, CD8 ^+^ cells were removed with Dynabeads (Invitrogen, 11147D). The cells were suspended (1×10 ^6^ cells/ml) in RPMI-1640 supplemented with L-glutamine, penicillin/streptomycin and 10% fetal bovine serum, and incubated in 5% CO _2_ at 37°C overnight.

HTLV-1-infected T cell clones ^4^ were maintained in RPMI-1640 (Sigma, R0883) supplemented with L-glutamine, penicillin/streptomycin and 20% fetal bovine serum (Gibco, 10500-064) in 5% CO _2_ at 37°C. IL-2 (Miltenyi Biotec, 130-097-745) was supplemented (100 unit/ml) into the culture twice a week. Raltegravir (Selleck Chemicals, MK-0518) was used at the concentration of 10 μM throughout the culture in order to prevent secondary infection.

### Flow cytometry and cell sorting

**Figure 2.**
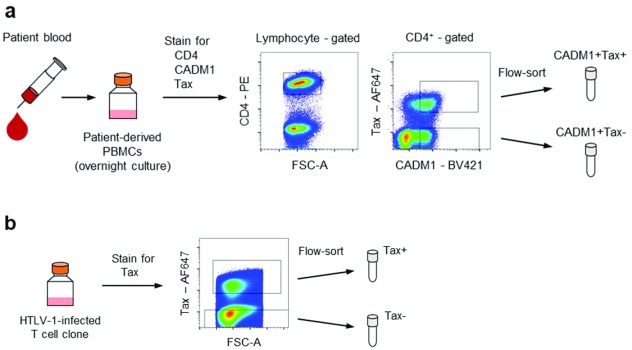
Overview of the cell preparations. ( **a**) Preparation of Tax ^+^ and Tax ^–^ populations from PBMCs obtained from HTLV-1-infected patients. PBMCs were stained for CD4, CADM1 and Tax after overnight culture. Tax ^+^ and Tax ^–^ fractions were collected from the CADM1 ^+^ population. ( **b**) Preparation of the Tax ^+^ and Tax ^–^ populations from HTLV-1-infected T cell clones. HTLV-1-infected T cell clones were stained for intracellular Tax and sorted according to Tax expression.

PBMCs were stained for surface markers CD4 and CADM1 following LIVE/DEAD cell staining (Invitrogen, L34976). Then the viral protein Tax was stained intracellularly with Foxp3 staining kit (eBioscience, 00-5523-00) ( Figure 2a). CADM1 staining was included in order to obtain an equivalent number of HTLV-1-infected cells in the Tax ^–^ population ^20^. HTLV-1-infected T cell clones were stained with LIVE/DEAD and anti-Tax antibody ( Figure 2b). The antibodies used were: mouse anti-CD4-PE (clone RPA-T4; BioLegend, 300507; concentration used, 0.8 μg/ml); chicken anti-CADM1-biotin (clone 3E1; MBL, CM004-6; 20 μg/ml) in combination with SA-BV421 (BioLegend, 405226; 1 μg/ml); mouse anti-Tax-Cy5 or anti-Tax-AF647 (clone LT-4; 0.4 μg/ml) ^21^. Cell sorting was carried out with a BD FACSAria III.

### Chromatin immunoprecipitation

Chromatin was sonicated and sheared following cell and nuclear lysis. Sheared chromatin was incubated with each of the following antibodies: rabbit polyclonal anti-H3K4me3 (Millipore, 07-473; 2 μg per assay), rabbit polyclonal anti-H3K9Ac (Millipore, 17-658; 2 μg), mouse anti-H3K27Ac (clone CMA309; Millipore, 17-683; 2 μg), rabbit polyclonal anti-H3K36me3 (Abcam, ab9050; 2 μg), rabbit polyclonal anti-CTCF (Millipore, 07-729; 2 μg) and normal rabbit IgG (Millipore, PP64B attached to 17-658; 2 μg). The resulting immune complexes were precipitated with Protein A+G magnetic beads (Millipore, 16-663). After washing the beads, the DNA fragments were recovered and purified.

### Library preparation and enrichment for the proviral DNA fragments

Adaptor DNA was attached to the fragments obtained from chromatin immunoprecipitation using NEBNext Ultra II DNA Library Prep Kit (NEB, E76455). Then DNA library fragments were enriched for the HTLV-1 proviral sequence by probe capture hybridization ^22^. Namely, DNA fragments were incubated in hybridization buffer (NimbleGen, 05634261001) at 65°C for 4 hours with human Cot-1 DNA (Invitrogen, 15279-011) and biotinylated hybridization probes complementary to the HTLV-1 provirus sequence. After hybridization, the probes and associated DNA library fragments were recovered with Streptavidin beads (Invitrogen, 65305), and the DNA fragments were PCR-amplified using NEBNext High-Fidelity 2X PCR Master Mix (NEB, M0541) with primers P5 (5′-AAT GAT ACG GCG ACC ACC GA-3′) and P7 (5′-CAA GCA GAA GAC GGC ATA CGA-3′) to a concentration of the order of 10 nM (98°C for 45 sec; varying cycles of 98°C for 15sec, 65°C for 30 sec and 72°C for 30 sec; 72°C for 1 min).

### High-throughput sequencing

The DNA library fragments were sequenced with Miseq Reagent Kit v3 (150 cycles) (Illumina).

All reads obtained from an HTLV-1 clone TBX4B were aligned to an HTLV-1 reference genome J02029 (ref. 23). The reads that overlapped either end of the provirus (J02029) were used to identify the host genomic sequence flanking the provirus in TBX4B (hg38 chr22: 43,927,318). The sequence around the provirus insertion site (TBX4B) was appended to the provirus (644 bp to the upstream and 632 bp to the downstream of the provirus sequence J02029) to create a custom reference for TBX4B. The J02029 reference was also used to align the reads obtained from PBMCs and other HTLV-1-infected T cell clones.

Paired 75 bp reads were aligned to the respective reference genome with BWA ^24^. Paired reads were kept if they were aligned within 80-800 nucleotides. PCR duplications were removed with Picard 2.6.0. The data was converted into the bedgraph format with bedtools ^25^, and visualised with the R Bioconductor package Sushi ^26^.

### Methylated DNA immunoprecipitation

DNA was extracted from fixed and flow-sorted HTLV-1-infected cells with the DNA FFPE Tissue Kit (Qiagen, 56404). DNA was sheared by sonication (Covaris) to obtain 200-600 bp fragments. Fragments containing methylated DNA were precipitated using MethylCollector Ultra Kit (Active Motif, 55005) with Low Salt Binding Buffer supplied by the manufacturer. The fragments were purified from either precipitated or unbound fraction with MinElute PCR purification Kit (Qiagen, 28004), and eluted into the same volume. For each locus in the HTLV-1 provirus indicated in Figure 4a, the abundance of precipitated fragments relative to the one from the unbound fraction was obtained by qPCR (95°C for 20 sec; 50 cycles of 95°C for 1 sec and 60°C for 20 sec) with the delta Ct method. The fraction size of captured fragments (%total) was calculated by [B]/([B]+[U]) × 100, where [B] and [U] denote the relative abundance of precipitated and unbound fragments, respectively. PCR primers used are listed in supplementary material ( Supplementary Table 1; Supplementary File 1).

### Bisulfite treatment and DNA sequencing

DNA was extracted from fixed and flow-sorted HTLV-1-infected cells with the DNA FFPE Tissue Kit (Qiagen, 56404). Purified DNA was subject to bisulfite treatment with EpiTect Bisulfite Kit (Qiagen, 59104). A nested PCR was performed with FastStart Taq DNA Polymerase (Roche, 04738357001) to amplify the regions indicated in Figure 5b. PCR conditions were as follows: 95°C for 5 min; 95°C for 30 sec, annealing temperature for 30 sec and 72°C for 30 sec (40 cycles); and 72°C for 2 min. The primer sequences and annealing temperatures are shown in the supplementary material ( Supplementary Table 2; Supplementary File 1). The PCR products were cloned into pGEM-T Easy (Promega) and Sanger-sequenced (GATC Biotech).

### CRISPR/Cas9 and cell cloning

Ribonucleoprotein (RNP) complex transfection was used ^27^. Namely, 2 μl of recombinant Cas9 protein (2.5 μg/μl) (PNA Bio, CP02) and 0.5 μl of *in vitro*-synthesised guide RNA (3 μg/μl) (Agilent, 5190-7706; DNA template, 5′-AAG CAC CGA CTC GGT GCC ACT TTT TCA AGT TGA TAA CGG ACT AGC CTT ATT TTA ACT TGC TAT GCT TTT CAG CAT AGC TCT AAA ACC GCG AGG TGG CGC TTT CTC CTA TAG TGA GTC GTA TTA CAT CG-3′) as well as 1 μl of homologous DNA repair template (100 μM) (5′-AGG AAG CTG TGC TTG ACG GTT TGC TAT CCT TAG AAG AGG AAA GCC GCG GCC GGC TGC GAC GGG GCC CTC CAG GGG AGA AAG CCC CGC CAA GAG GTG AAA CGC ATC GTG ATC GGC AGC GAC GGG CTG AGG AGA AGA GGA AGC GAA AAA AAG AGC GGG AGA AAG AGG AGG AAA AGC AG-3′) (Integrated DNA Technologies) were combined with 8 μl of R resuspension buffer from Neon transfection system (Invitrogen). Cells (5 × 10 ^5^) were suspended in buffer containing RNP complex and transfection was performed with Neon (Invitrogen) (1600 V, 10 msec and 3 pulses). When cells had recovered at around a week after transfection, DNA was extracted with DNA extraction solution (Epicentre, QE09050). The absolute copy numbers of *gag* and the mutant proviral sequence of the CTCF site in the DNA were quantified by qPCR (50°C for 2 min; 95°C for 10 min; 50 cycles of 95°C for 15 sec and 60°C for 1 min) with respective standard curves, in order to estimate the frequency of mutant cells in the culture. Primers and probes for *gag* (forward, 5′-TTA TGC AGA CCA TCC GGC TT-3′; reverse, 5′-TAT CTA GCT GCT GGT GAT GGA G-3′; probe, 5′-CGG TGC AGC AGT TTG ACC CCA CTG C-3′) and mutant CTCF-binding site (forward, 5′-CTG CTT TCT CCG GGC GAC or CTG CTT TCT CCG GGC AAA G-3′; reverse, 5′-AGC CCC GCC AAG AGG T-3′; probe, 5′-AAC GCA TCG TGA TCG GCA GCG AC-3′) were used. Mutants were detected at a frequency of 1.6% to 3.1% in each of 4 HTLV-1-infected T cell clones applied. Cells were subcloned in order to isolate mutant cells by either limiting dilution or flow-sorting (BD FACSAria III) under Containment Level 3 conditions. Subclones were screened for the mutant proviral sequence of the CTCF site by PCR (95°C for 20 sec; 40 cycles of 95°C for 1 sec and 60°C for 20 sec) with a mutation-specific primer indicated above. The DNA sequence of the putative mutants was confirmed by Sanger sequencing (GATC Biotech).

### Single-molecule RNA-FISH

HTLV-1-infected T cell clones were subjected to single-molecule RNA-FISH, targeting the plus- or minus-strand transcripts of HTLV-1, following the protocol described previously ^17^. The coverslips were imaged with an Olympus IX70 inverted widefield microscope with a 100× 1.35NA UPlanApo oil objective lens, a Spectra Light Engine illumination source (Lumencor) and an ORCA-Flash 4.0 V2 digital CMOS camera (Hamamatsu).

### Droplet digital PCR

The DNA samples obtained by ChIP using anti-CTCF antibody or normal rabbit IgG described above were quantified by droplet digital PCR. The reaction was set up using ddPCR Supermix (Bio-Rad, 186-3023) containing 750 nM each of forward and reverse primers and 250 nM probe to detect the HTLV-1 CTCF-binding site and TC-1 locus. Primers and probe for the HTLV-1 CTCF-binding site are listed in Supplementary File 1 (Target pX in Table 1). For TC-1 locus: forward primer, 5′-TCT CCA GCA CTT CTT GCT CA-3′; reverse primer, 5′-TGG GAT GGC TAA CCT GTT GT-3′; probe, 5′-TCT CTG CTG CTC CCA GGC GGC-3′. Droplets were generated with QX200 Droplet Generator (Bio-Rad). PCR was performed (95°C for 10 min; 40 cycles of 94°C for 30 sec and 60°C for 1 min; 98°C for 10 min) with C1000 Touch thermal cycler (Bio-Rad). Fluorescent signal was detected using QX200 Droplet Reader (Bio-Rad).

## Results

To identify the epigenetic modifications associated with transcriptional activity in the provirus, we sorted the cells based on Tax protein expression and performed ChIP and DNA methylation analyses for each fraction ( Figure 2) unless stated otherwise.

In the descriptions of the ChIP-seq data, we use the terms “5′ LTR junction” and “3′ LTR junction” to denote the regions of the HTLV-1 provirus adjoining the 5′ LTR and the 3′ LTR, respectively.

### Histone modifications are strongly associated with plus-strand transcription

**Figure 3.**
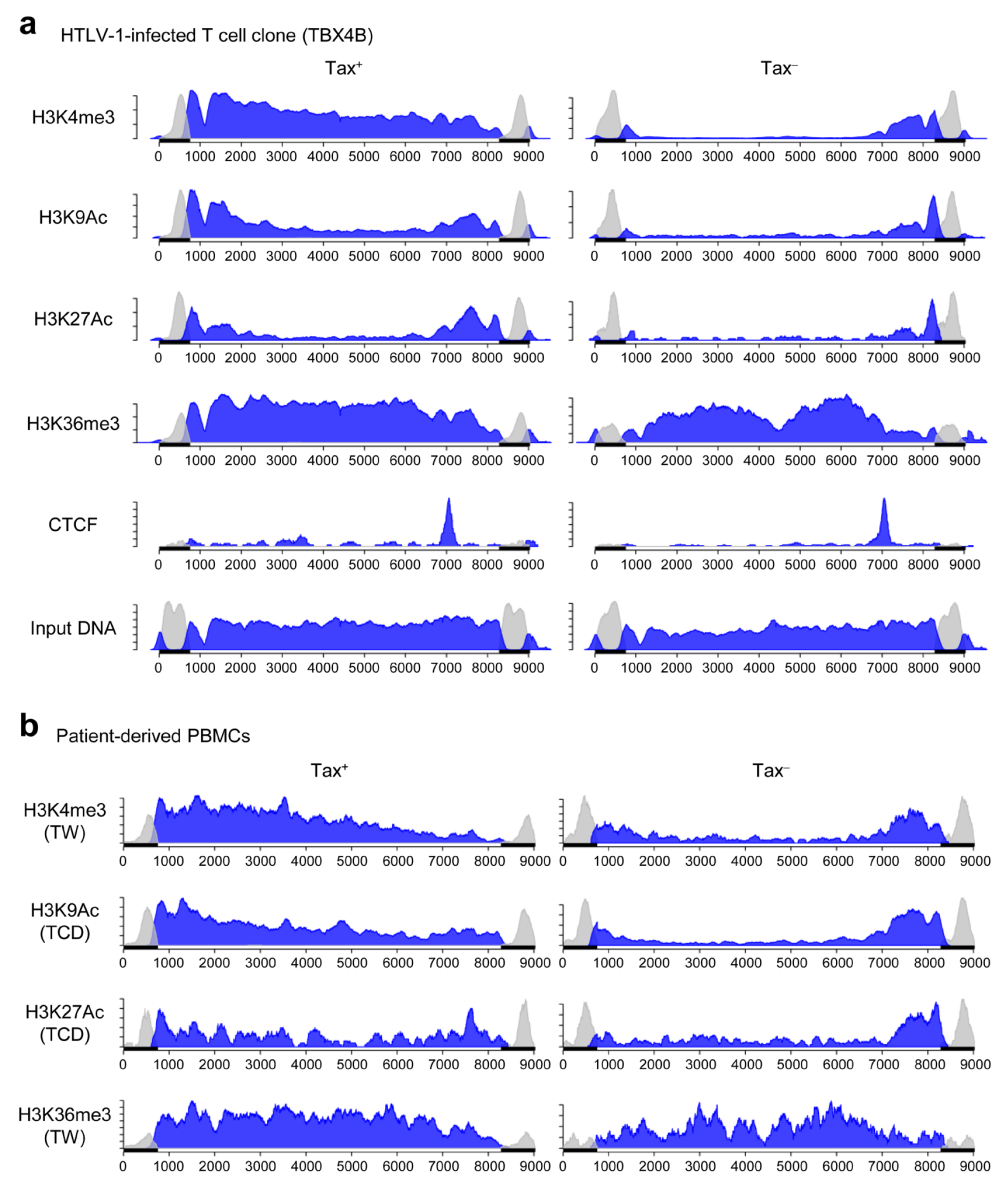
Histone modifications and CTCF-binding in the HTLV-1 provirus. Chromatin immunoprecipitation (ChIP) was used to identify ( **a**) histone modifications and CTCF-binding in the Tax ^+^ and Tax ^–^ populations from an HTLV-1-infected T cell clone (TBX4B); and ( **b**) histone modifications in the CADM1 ^+^Tax ^+^ and CADM1 ^+^Tax ^–^ populations from PBMCs obtained from HTLV-1-infected patients (Patients TW and TCD). The horizontal axis indicates the nucleotide position in the full-length HTLV-1 provirus (J02029), and the vertical axis the read depth (arbitrary units). The reads that aligned within either one of the LTRs are greyed out. The black bars on the horizontal axis indicates the LTRs.

We first studied *in vitro* HTLV-1-infected T cell clones, because the cells in each clone share the same provirus insertion site, so minimizing effects due to heterogeneity in the host genomic environment of the provirus. We performed ChIP on the Tax ^+^ and Tax ^–^ populations from one of the HTLV-1-infected T cell clones to identify the histone modifications in the HTLV-1 provirus ( Figure 3a). The HTLV-1 provirus was marked with H3K4me3 from the 5′ LTR junction through to the 3′ LTR junction in the Tax ^+^ population. Substantial signals from other histone marks H3K9Ac and H3K27Ac were also detected in the 5′ LTR junction and *gag* in the Tax ^+^ population. These histone marks are associated with promoters and enhancers with active transcription ^28^ In the pX region and the 3′ LTR junction, these three histone marks (H3K4me3, H3K9Ac and H3K27Ac) were constantly detected, regardless of Tax expression. In particular, H3K4me3 was more highly enriched in those regions in the Tax ^–^ population ( Figure 3a and Supplementary Figure 1a), consistent with the observation of differential expression of the plus- and minus-strands. A similar pattern was observed in another *in vitro* HTLV-1-infected T cell clone (11.65) ( Supplementary Figure 1b). The small signal of H3K4me3 in the Tax ^–^ population ( Figure 3a and Supplementary Figure 1b) is a characteristic feature of a poised promoter.

**Figure 4.**
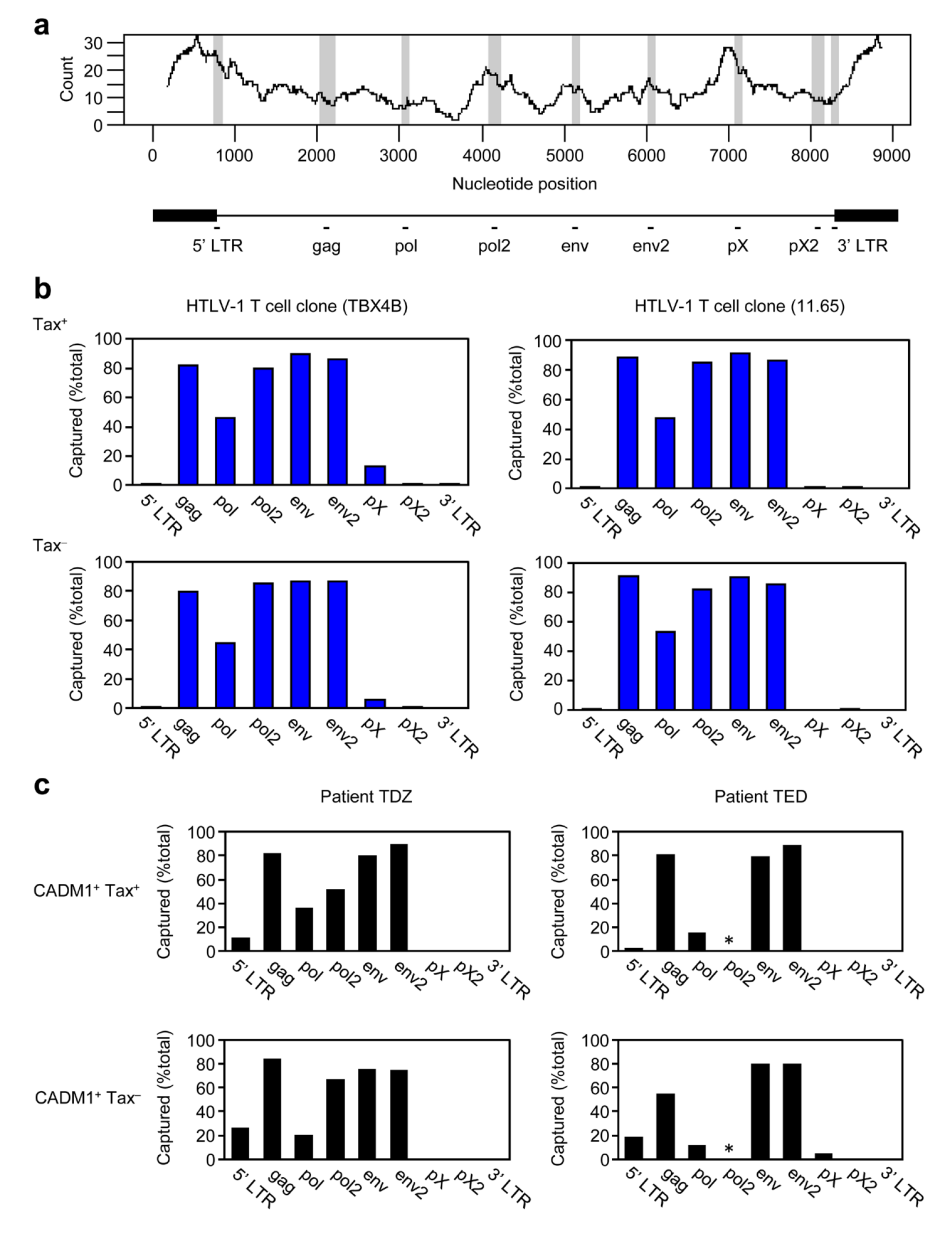
DNA methylation across the body of the HTLV-1 provirus. ( **a**) Upper panel: count of CpG dinucleotides in a window of 350 bp in the HTLV-1 reference genome (L36905). Lower panel: schematic diagram of HTLV-1 provirus indicating the two LTRs and the 9 loci examined by MeDIP. ( **b**) DNA methylation on the HTLV-1 provirus in the Tax ^+^ and Tax ^–^ populations from two HTLV-1-infected T cell clones (Clones TBX4B and 11.65). ( **c**) DNA methylation on the HTLV-1 provirus in the CADM1 ^+^Tax ^+^ and CADM1 ^+^Tax ^–^ populations in PBMCs from two unrelated individuals (Patients TDZ and TED). The asterisk (*) indicates that the PCR failed to amplify.

**Figure 5.**
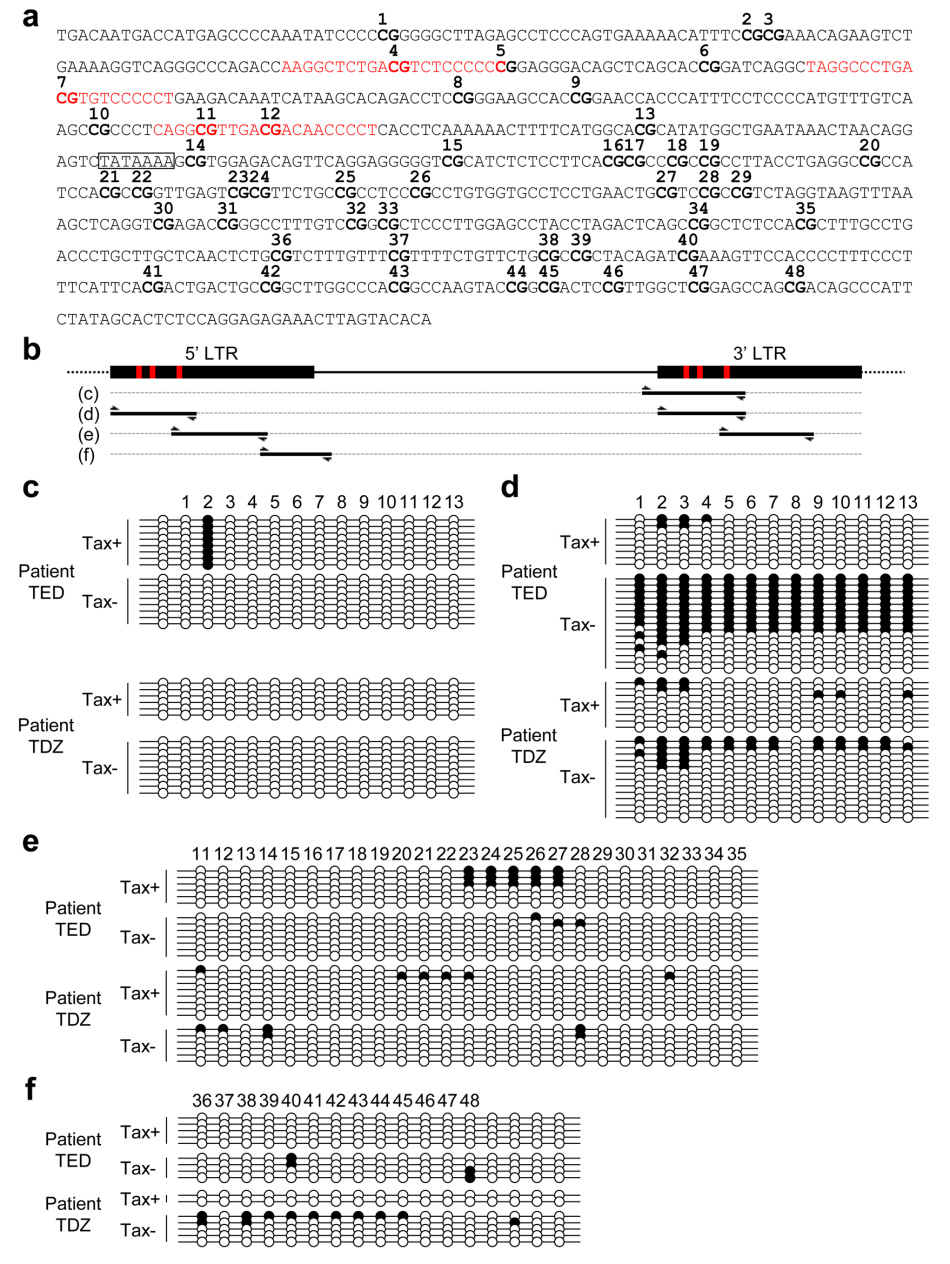
DNA methylation in the HTLV-1 LTR of patient-derived PBMCs. ( **a**) The HTLV-1 LTR sequence (Accession no. L36905) with CpG dinucleotides highlighted in bold. The three TREs are coloured in red; the TATA box is indicated in the rectangle. ( **b**) Schematic diagram of HTLV-1 provirus and the regions amplified with indicated sets of primers for bisulfite-sequencing. Sequencing results for each region are shown in the corresponding panels ( **c**– **f**). The three TREs are indicated by red bars. ( **c**– **f**) Schematic representation of DNA methylation for each clone sequenced. Open circles indicate unmethylated cytosine; closed circles methylated cytosine. The numbers indicate the corresponding CpG sites in panel ( **a**).

Next, we examined patient-derived PBMCs (patient TW for H3K4me3 and H3K36me3; patient TCD for H3K9Ac and H3K27Ac) after overnight culture ( Figure 3b). The pattern of histone modifications was largely the same as that observed in HTLV-1 clones, in that there was a much stronger signal for H3K4me3, H3K9Ac and H3K27Ac from the 5′ LTR junction in the Tax ^+^ population, and those marks appeared persistent in the 3′ LTR junction regardless of viral reactivation.

Because we observed a similar pattern of histone marks in two independent HTLV-1-infected T cell clones with distinct provirus insertion sites, as well as in PBMCs with polyclonal insertion sites, the pattern of histone marks that we observed is not likely to be dependent on the host genomic environment of the provirus, but rather is a feature intrinsic to the HTLV-1 provirus. Two observations indicate that the changes in the observed pattern of histone marks are rapid and reversible: first, HTLV-1 reactivation takes place within a few hours of culture in patient-derived PBMCs ^15, 16^; second, the HTLV-1-infected T cell clones demonstrate intermittent bursts of Tax expression ^17, 29^. Currently, there is no means of separating HBZ ^+^ and HBZ ^–^ populations. However, we anticipate that the changes in histone marks in the 3′ LTR junction are likely to be small, and the minus-strand expression is much more constant than the plus-strand transcription ^17^.

Confirming our previous report ^19^, we observed binding of the host protein CTCF in the HTLV-1 provirus ( Figure 3a) at the boundary in the pX region where the histone marks H3K4me3, H3K9Ac and H3K27Ac are confined to the 3′ end of the provirus. We hypothesized that the host protein CTCF regulates the epigenetic modifications: we discuss this hypothesis below.

### DNA in the pX and 3′ LTR region is left unmethylated regardless of the plus-strand expression

Next we wished to examine if DNA methylation in the provirus also correlates with HTLV-1 proviral transcription, as we saw in the histone marks above. We performed methylated DNA immunoprecipitation (MeDIP) and examined 9 loci across the HTLV-1 provirus by qPCR ( Figure 4a). In the HTLV-1-infected T cell clones (TBX4B and 11.65) ( Figure 4b), regardless of Tax expression, DNA in the HTLV-1 provirus was methylated in the *gag*, *pol* and *env* regions, whereas the pX and 3′ LTR regions were not methylated. The signal from the *pol* locus was lower than the other sites, perhaps because there are fewer CpG sites in this locus ( Figure 4a). Note that, on the contrary, the pX region was hypomethylated ( Figure 4b) despite the higher frequency of CpG in this region ( Figure 4a).

We also examined patient-derived PBMCs (Patients TDZ and TED) ( Figure 4c) cultured overnight. Regardless of the plus-strand reactivation, DNA in the region from *gag* to *env* was heavily methylated. On the other hand, again, the pX and 3′ LTR regions were not methylated. The *pol2* site in Patient TED was not detected, perhaps because of sequence polymorphism in HTLV-1.

Regardless of the plus-strand expression, the pattern of DNA methylation in the body of the HTLV-1 provirus was essentially the same: that is, largely methylated but for the pX and 3′ LTR regions. The CTCF-binding site in the HTLV-1 provirus is in the pX region ( Figure 3a): as previously reported ^19^, this CTCF-binding site is situated at the observed border of DNA methylation ( Figure 4b and c).

### DNA hypomethylation in the plus-strand promoter is a prerequisite for viral reactivation

To investigate further the putative link between the DNA methylation and viral expression, we examined the HTLV-1 plus-strand promoter region. In the first half of the HTLV-1 LTR, there are three Tax-response elements (TREs), among other transcription factor binding sites, upstream of the TATA box ( Figure 5a). The TREs serve as the promoter for plus-strand transcription. We used fresh, patient-derived PBMCs, as they are most likely to maintain the DNA methylation pattern *in vivo*. There are 48 CpG sites in the HTLV-1 LTR (Accession number L36905) ( Figure 5a). Because the two HTLV-1 LTRs have an identical sequence, it is not possible to specifically amplify one of the LTRs for bisulfite-sequencing. Therefore we took the approach of Koiwa *et al.* ^30^, as follows.

First, the 5′ half of the 3′ LTR was specifically amplified as is indicated in Figure 5b by line (c). None of the fragments were methylated, except for position 2 in the Tax ^+^ population from Patient TED ( Figure 5c). (Here, we designate fragments with a few positions methylated as hypomethylated.) This observation was consistent with what we observed in the MeDIP assay targeting the 3′ LTR ( Figure 4c). Next, the same part of the HTLV-1 LTR was amplified from either the 5′ LTR or 3′ LTR, as is indicated by line (d) in Figure 5b. We assume that fragments are amplified from the 5′ LTR and 3′ LTR with equal efficiency. The results ( Figure 5c) showed that most fragments from the 3′ LTR were hypomethylated. DNA methylation in the three TREs (position 4–12) was observed exclusively in the Tax ^–^ population ( Figure 5d). We infer that, in most of the Tax ^–^ cells in Patient TED, the 5′ LTR promoter is methylated. In the Tax ^–^ population from Patient TDZ, DNA methylation in the 5′ LTR appeared less frequent, which leaves open the possibility that the DNA methylation is not the determining factor for viral latency. On the other hand, the 5′ LTR promoter was likely to be hypomethylated when Tax was expressed in both patients. Then we expanded the search area for the DNA methylation further into the LTR, as indicated by lines (e) and (f) ( Figure 5b). In general, CpG sites after position 13, and hence downstream of the TREs, were not heavily methylated whether in the 5′ LTR or 3′ LTR, and regardless of Tax expression ( Figure 5e). Similarly, the 3′ end of the 5′ LTR was not methylated ( Figure 5f), with an apparent exception in the Tax ^–^ population from Patient TDZ.

To summarise, DNA methylation in the HTLV-1 LTR is confined within the first half of the LTR, which contains three TREs. DNA is not methylated when the plus strand is expressed; however, it is not yet clear whether DNA methylation alone is sufficient to explain proviral latency. In HTLV-1-infected T cell clones cultured *in vitro*, the 5′ LTR promoter was hardly methylated, regardless of whether Tax is expressed at a given time ( Supplementary Figure 2a and b). The DNA methylation pattern was much less variable in the HTLV-1-infected T cell clones than in the PBMCs: each clone is derived from a single cell, so every cell in that clone carries the HTLV-1 provirus in the same genomic site. Nevertheless, this reinforces the notion that DNA methylation is not the sole factor that suppresses, even if temporarily, the viral transcription.

### Altering the sequence of CTCF-binding site in the HTLV-1 provirus by Cas9/gRNA RNP transfection

**Figure 6.**
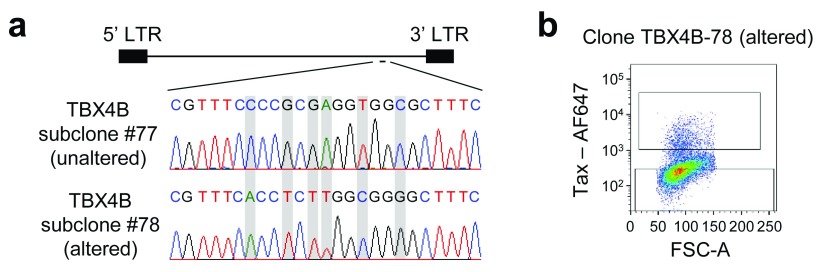
Alteration of the CTCF-binding site in the provirus in HTLV-1-infected T cell clones. ( **a**) The sequence of the CTCF-binding site in the HTLV-1 provirus. The upper panel is from a subclone with the sequence unchanged, and the lower panel from a subclone in which the sequence was altered by CRISPR/Cas9 modification. ( **b**) Flow cytometric analysis of the mutated clone after staining for intracellular Tax protein.

We observed above that the CTCF-binding site in the HTLV-1 provirus coincides with the apparent boundary of epigenetic modifications (i.e. both the histone marks and DNA methylation) in the provirus. Considering the known functions of CTCF, which include transcription regulation and the formation of an epigenetic barrier, we hypothesized that CTCF in the pX region of the HTLV-1 provirus controls the epigenetic modifications and viral transcription. To test this hypothesis, we applied CRISPR/Cas9 modification to alter the sequence of the CTCF-binding site, using ribonucleoprotein complex transfection ^27^. We isolated mutant cells by subcloning and confirmed that the sequence at the CTCF-binding site had been correctly altered ( Figure 6a). We previously showed that this alteration is sufficient to abrogate CTCF binding to the provirus ^19^. Nevertheless, we observed that the mutant clones still expressed Tax ( Figure 6b). We therefore set out to examine the epigenetic modifications in ΔCTCF-binding clones as described below.

### The epigenetic modifications in the HTLV-1 provirus are CTCF-independent

**Figure 7.**
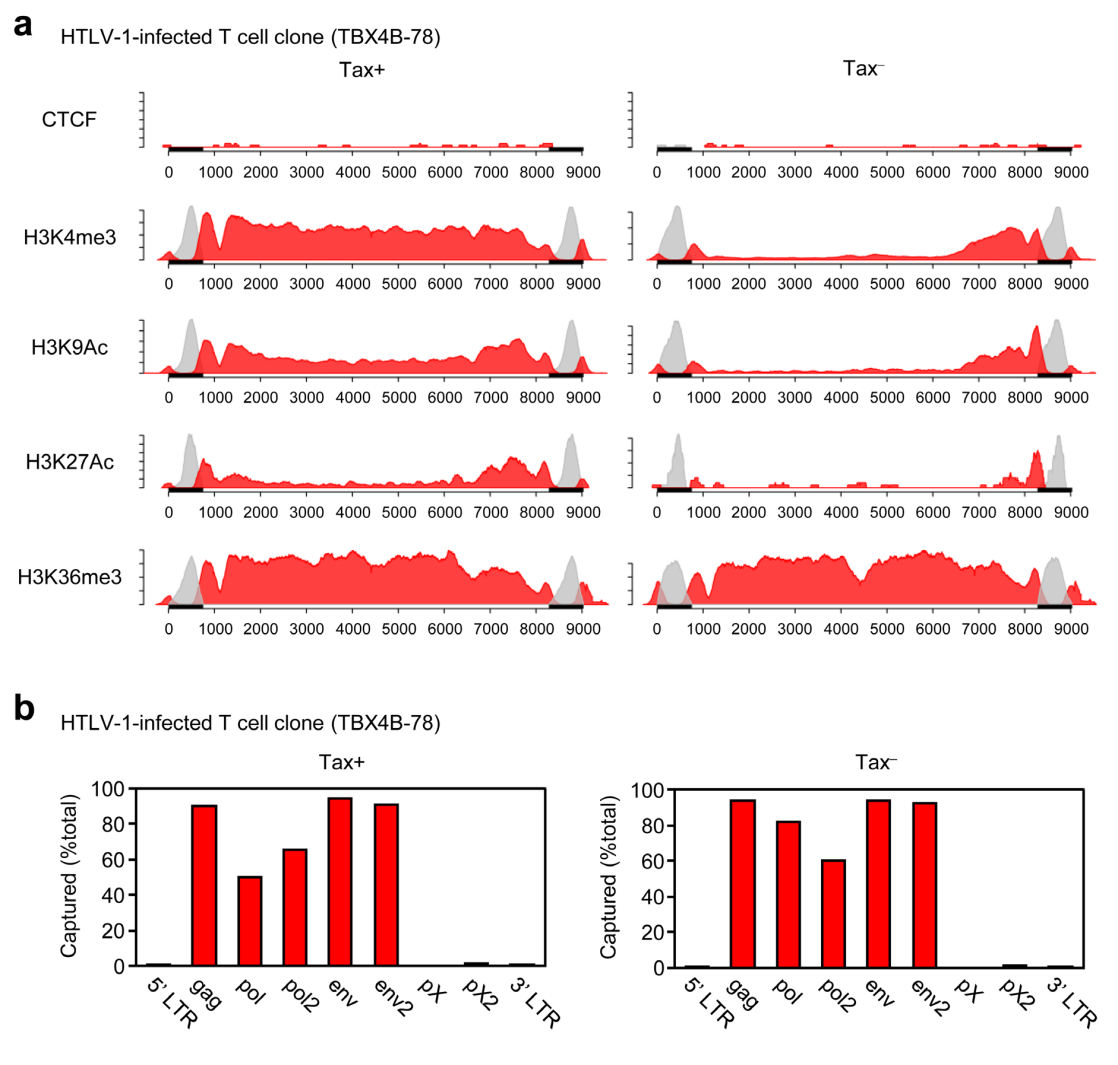
Epigenetic modifications in the HTLV-1 provirus lacking CTCF binding. ( **a**) Histone modifications in the Tax ^+^ and Tax ^–^ populations of the altered HTLV-1-infected T cell clone (Subclone #78 of TBX4B). ( **b**) DNA methylation in the body of the provirus in TBX4B-78. Note the similarity to the profiles of epigenetic modifications in the wild-type TBX4B ( Figure 3).

We examined the epigenetic modifications in a ΔCTCF-binding clone (TBX4B-78). First, we confirmed that CTCF was no longer detected in either the Tax ^+^ or Tax ^–^ populations ( Figure 7a). The pattern of the histone marks ( Figure 7a) was largely the same as that in the parental clone ( Figure 3a), in that the changes in H3K4me3, H3K9Ac and H3K27Ac were associated with Tax expression, and were stable downstream of nucleotide ~7000 (CTCF-binding site). The profile of H3K36me3 showed a reproducible small dip in the middle of the provirus specifically in the Tax ^–^ cells. Similarly, DNA methylation in the body of the provirus ( Figure 7b) was not distinct from that in the parental clone ( Figure 4b): *gag*, *pol* and *env* were largely methylated, whereas the pX and 3′ LTR regions were not (again downstream of the CTCF-binding site). The 5′ LTR promoter was also hypomethylated, regardless of Tax expression in ΔCTCF-binding clones ( Supplementary Figure 2a and c). These results are inconsistent with the idea that CTCF imposes an epigenetic border in the HTLV-1 provirus and regulates the distinct modifications around the pX region.

Since the technique of ChIP-sequencing coupled with probe capture hybridization lacks precise quantification, we went on to quantify the kinetics of the plus- and minus-strand transcription of HTLV-1 more precisely, using our previously described protocol of single-molecule RNA-FISH, to detect any possible impact of CTCF-binding on HTLV-1 transcription.

### Removal of CTCF does not affect the kinetics of the plus- and minus-strand expression *in vitro*

**Figure 8.**
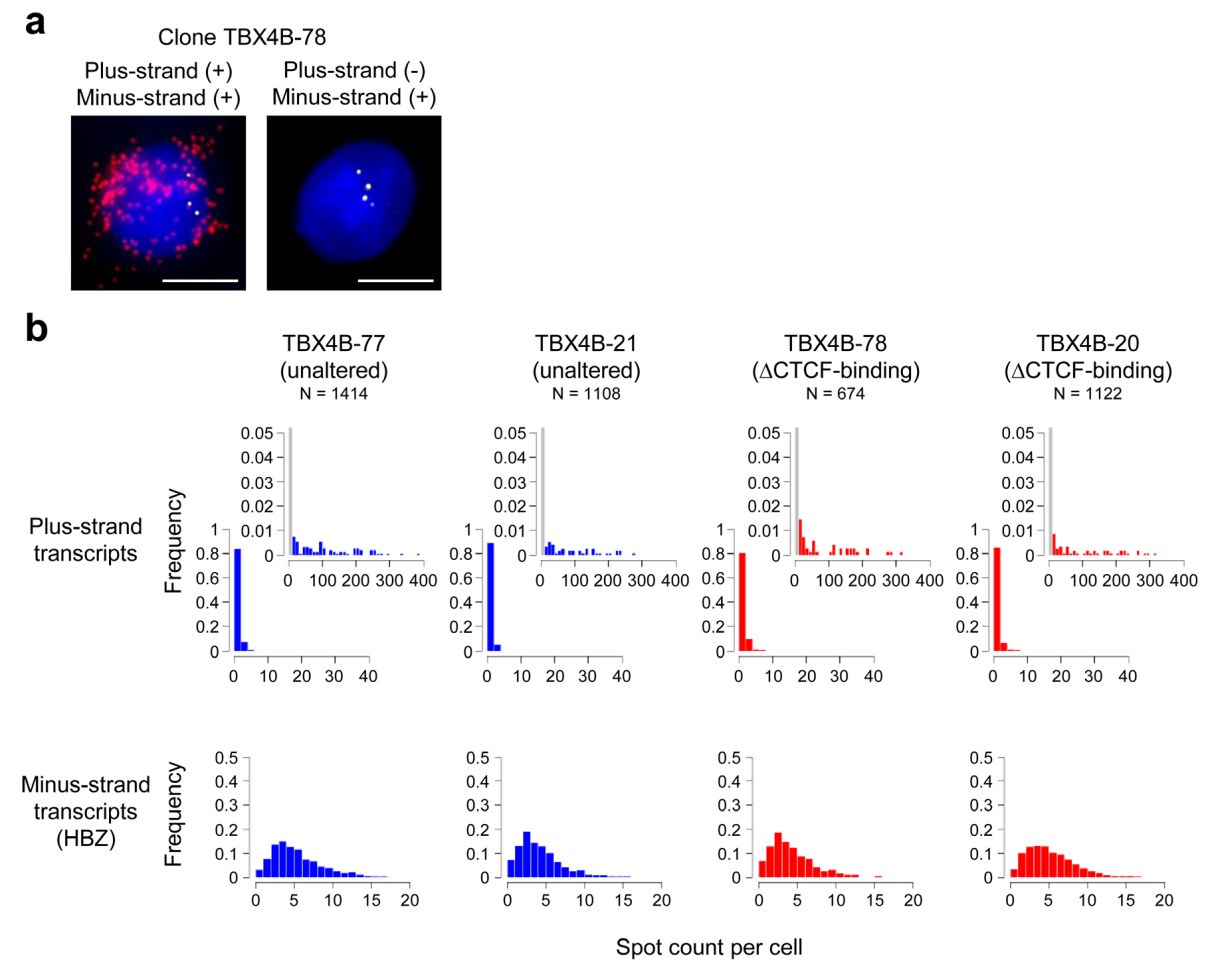
Kinetics of the plus- and minus-strand transcription in HTLV-1-infected T cell clones. ( **a**) Representative images of HTLV-1 transcripts by single-molecule RNA-FISH (maximum-projection of Z-stacks). Red spots indicate the plus-strand transcripts, and yellow spots the minus-strand transcripts. Blue indicates the DAPI-stained nucleus. Plus- and minus-signs in brackets indicate respectively the presence or absence of the mRNA. Scale bar (white) = 5 µm. ( **b**) Spot counts of the plus-strand (upper row) and the minus-strand transcripts (lower row) respectively in the unaltered and ΔCTCF-binding subclones. The insets in the upper row capture low-frequency events on a magnified y-axis. The bar in the first bin in the insets is greyed out because it is out of scale.

We have recently reported that the HTLV-1 plus-strand transcription shows periods of transient, rapid and intense spontaneous activity, often referred to as a transcriptional burst, whereas the minus-strand expression is relatively stable ^17^. To test whether CTCF has an impact on the pattern of the plus- and minus-strand expression, we performed single-molecule RNA-FISH on the ΔCTCF-binding clones. Representative images are shown in Figure 8a. As reported in our recent publication ^17^, a limited fraction of cells had a large number of plus-strand transcripts, and the remaining cells were negative. On the other hand, minus-strand transcripts were present in most cells. The number of transcripts per cell in the ΔCTCF-binding clones is presented in Figure 8b. The distribution of the plus-strand transcripts was indistinguishable between the ΔCTCF-binding and unmodified subclones from TBX4B. This trend was also the case for the minus-strand transcript ( *HBZ*). We confirmed this result with another HTLV-1-infected clone (11.50) ( Supplementary Figure 3). This result shows that the removal of CTCF did not affect the transcriptional activity of HTLV-1. Therefore, it is unlikely that any difference in the degree of epigenetic modifications between the CTCF-mutant and parental HTLV-1-infected T cell clones has a significant impact on the transcriptional activity of HTLV-1.

### CTCF occupancy does not predict the viral reactivation in patient-derived PBMCs

The putative impact of CTCF in the provirus was tested above on the HTLV-1-infected T cell clones. These clones were initially isolated and expanded from PBMCs of HTLV-1-infected individuals, and show robust growth *in vitro*. It is likely that they were selected for strong *in vitro* growth, and so may have diverged phenotypically from the PBMCs *in vivo*; such differences could conceivably affect HTLV-1 transcription. Therefore, we wished to test the putative association between CTCF-binding and viral reactivation using PBMCs from HTLV-1-infected subjects.

**Figure 9.**
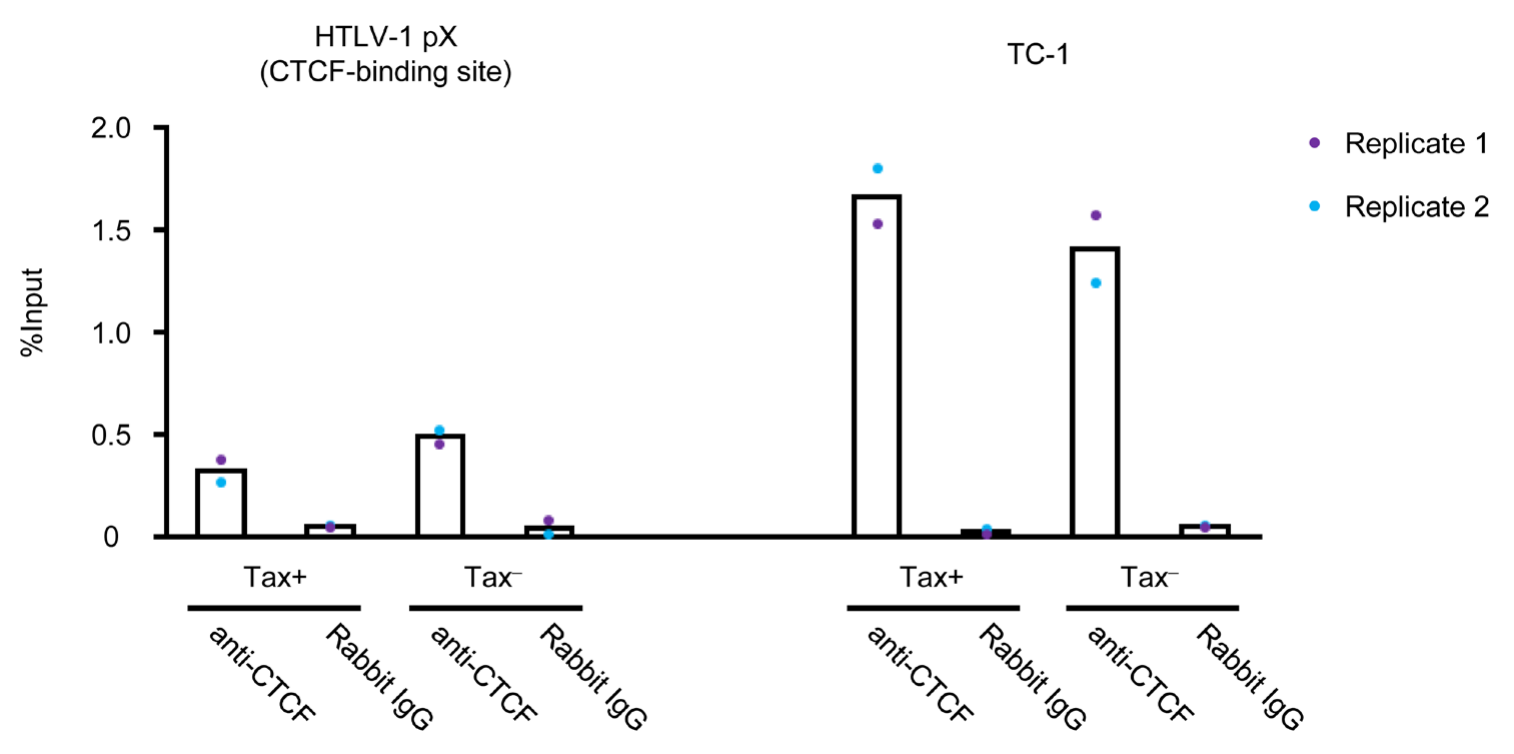
CTCF occupancy in the HTLV-1 provirus in patient-derived PBMCs. CTCF occupancy was examined by droplet digital PCR following ChIP for CTCF. The experiment was carried out on PBMCs after overnight incubation *in vitro*. Replicate 1 is obtained from pooled samples of 4 patients (TCR, TEJ, TED and TW) and Replicate 2 from 3 patients (TED, TCR and TEJ).

It is estimated ^31^ that there are tens of thousands of different HTLV-1-infected T cell clones in a typical HTLV-1-infected individual. Each clone carries a single copy of the provirus inserted in a unique location in the genome. Whether the viral reactivation takes place has a strong dependence on the genomic insertion site of the provirus ^32^. We asked if those clones that reactivate the plus-strand transcription have differential CTCF occupancy in the provirus from those that remain silent after the short-term culture. We performed a ChIP assay targeting CTCF on the Tax ^+^ and Tax ^–^ populations after overnight culture, and performed droplet digital PCR to quantify the CTCF occupancy. The results showed no measurable difference in CTCF-binding between the two populations ( Figure 9).

## Discussion

HTLV-1 has two identical LTRs, one at each end of the provirus, which serve as the promoters for the plus- and minus-strand transcription. One of the conundrums is how HTLV-1 keeps the plus-strand predominantly silenced while sparing the minus-strand expression *in vivo*. Attempts have been made to investigate the epigenetic modifications that differ between the 5′ LTR and 3′ LTR ^19, 30, 33, 34^. However, recent work by us ^17^ and others ^29^ shows that the HTLV-1 plus-strand transcription is highly variable over time. Thus, the epigenetic modifications we observe are an average of the two populations: one that is actively transcribing the plus strand, and the remaining cells that are not transcribing the plus strand at that time. Therefore in this study, we investigated what epigenetic modifications accompany the plus-strand transcription, by separating the two HTLV-1-infected populations.

Our results show that the changes in histone marks H3K4me3, H3K9Ac and H3K27Ac in PBMCs are specific to the Tax ^+^ population ( Figure 3b). These histone marks are generally accompanied by active transcription, which in the case of HTLV-1 begins within the first few hours of culturing PBMCs ^15, 16^. In *in vitro* HTLV-1-infected T cell clones, proviral transcription is not a one-off event: each cell appears to switch on and off the plus-strand transcription ^18^. We captured a snapshot of histone modifications when Tax is expressed ( Figure 3a). We conclude that the changes in histone modifications in HTLV-1 are highly dynamic: they are rapid and reversible.

DNA in the body of the HTLV-1 provirus is largely methylated except for the pX region and 3′ LTR ^34^. DNA methylation in the *gag*, *pol* and *env* regions has been considered as one of the mechanisms by which HTLV-1 maintains latency ^34^. However, in this study, we observed DNA methylation in the body of the provirus even in the Tax ^+^ population ( Figure 4c). Therefore, we conclude that DNA methylation in the gene body of the provirus has little impact on HTLV-1 transcription. HTLV-1 rapidly reactivates in *ex vivo* culture, but it is not yet known whether this is a physiological response or whether it results from a supraphysiological stress. It is possible, although we consider it unlikely, that the DNA methylation in the body of the provirus helps to maintain HTLV-1 latency *in vivo*.

DNA hypomethylation in the 5′ LTR promoter is associated with proviral transcription, as previously reported ^30^. We conclude that DNA hypomethylation in the plus-strand promoter is likely to be required for viral reactivation, consistent with the idea ^30^ that DNA methylation preferentially silences plus-strand expression *in vivo*. However, it is not proved whether DNA methylation is sufficient for HTLV-1 latency, because our results do not permit an accurate estimate of the frequency of methylation in the Tax ^–^ population. High-throughput sequencing for bisulfite-treated DNA is required to reveal the accurate view of DNA methylation associated with HTLV-1 latency. Nevertheless, assuming that DNA methylation is stable during the short-term culture of HTLV-1 ^35^, we propose the following: (1) there are two categories of HTLV-1-infected cells *in vivo* - those with the 5′ LTR promoter methylated and those in which it is hypomethylated; (2) viral reactivation is allowed only in the hypomethylated cells; and (3) the DNA methylation in the body of the provirus does not influence viral reactivation *ex vivo*. Whether proviral reactivation takes place depends strongly on the provirus insertion site ^32^. It is therefore possible that DNA methylation in the 5′ LTR promoter is related to the provirus insertion site.

Following the discovery that HTLV-1 binds CTCF ^19^, we hypothesized that CTCF imposes a boundary in the histone modifications and DNA methylation around the CTCF-binding site in the pX region. However, our results do not support the hypothesis that the pattern of epigenetic modifications in HTLV-1 depends directly on CTCF-binding. It is possible that changes in epigenetic modifications would take a longer time to become established after the provirus is mutated, or that CTCF imposes an epigenetic boundary at the pX region in the early stage of infection and becomes dispensable thereafter. However, the chromatin boundary in the pX region is not static: our observations indicate that, each time a cell goes through the cycle of plus-strand expression, the histone modifications change rapidly in the provirus, yet they always return to the marks present in the previous state of plus-strand expression, even without CTCF binding. We conclude that CTCF binding does not directly impose a barrier to the spread of these epigenetic modifications. Instead, it is possible that CTCF confers a benefit on HTLV-1 by making chromatin loops with the nearby host genome ^36^. However, the consequences of HTLV-1 inserting an ectopic CTCF-binding site in the host genome vary widely according to the genomic integration site: it remains to be seen whether there is an additional impact of CTCF binding to the HTLV-1 provirus that is consistent in all clones. It is also possible that CTCF confers a higher rate of HTLV-1 transmission and increases viral persistence *in vivo*. However, long-term animal model experiments may be necessary to test this hypothesis. It remains an open question what regulates the distinct epigenetic modifications observed around the pX region in HTLV-1.

## Data availability

The following datasets are available from Open Science Framework:

Dataset 1 (ChIP-seq on epigenetic marks):

https://doi.org/10.17605/OSF.IO/4Q9RY ^37^

Dataset 2 (image data on smFISH):

https://doi.org/10.17605/OSF.IO/4TBQY ^38^

Dataset 3 (qPCR and ddPCR data from ChIP and MeDIP):

https://doi.org/10.17605/OSF.IO/8M76K ^39^

Dataset 4 (Sanger sequencing data from bisulfite assay):

https://doi.org/10.17605/OSF.IO/D62H7 ^40^

These datasets are available under a CC0 1.0 Universal licence.

## Consent

Human subjects: All donors gave written informed consent in accordance with the Declaration of Helsinki to donate blood samples to the Communicable Diseases Research Tissue Bank, approved by the UK National Research Ethics Service (15/SC/0089).

## Supplementary material

Supplementary Figure 1: Histone modifications in HTLV-1 provirus

Supplementary Figure 2: DNA methylation in the 5′ LTR promoter in HTLV-1-infected clones

Supplementary Figure 3: Count of viral transcripts in an HTLV-1+ T cell clone

Supplementary File 1: File containing the following supplementary tables listing primers and probes used for PCR amplification.

Supplementary Table 1: PCR primers used for methylated DNA immunoprecipitation and qPCR.

Supplementary Table 2: PCR primers and annealing temperature to amplify bisulfite-treated HTLV-1 provirus.
