# Supplementary material for: Epigenetic changes around the pX region and spontaneous HTLV-1 transcription are CTCF-independent
Source: Wellcome Open Res. 2018 Dec 11;3:105. Originally published 2018 Aug 24. [Version 2] doi: 10.12688/wellcomeopenres.14741.2 (PMC6305241; doi:10.12688/wellcomeopenres.14741.2)
Supplement: Supplementary file 1 [file wellcomeopenres-3-16324-s0004.tgz › bc160e18-eca7-488c-af20-16709a7f80cf_revised_Supplementary_Figure_1.pdf]

Spontaneous HTLV-1 transcription and epigenetic changes around the pX region are CTCF-independent.

Miura M *et al*. *Wellcome Open Res* 2018

Supplementary material

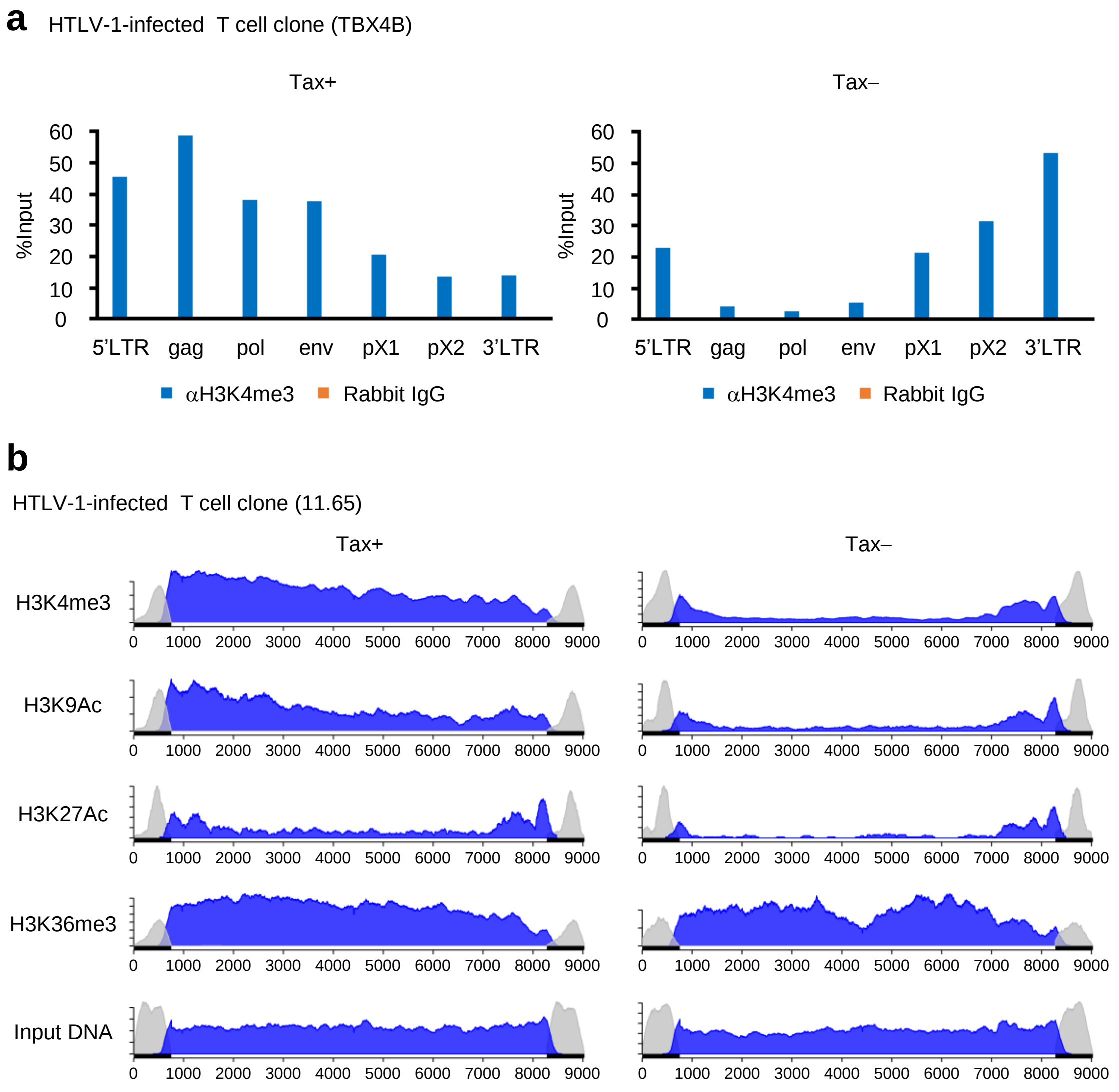

Supplementary Figure 1 Histone modifications in HTLV-1 provirus

(a) Histone modifications in each of the Tax$^+$ and Tax$^-$ populations from an HTLV-1-infected T cell clone (TBX4B). The provirus loci examined by qPCR on the horizontal axis correspond to the ones shown in Figure 4a. (b) Histone modifications in each of the Tax$^+$ and Tax$^-$ populations from an HTLV-1-infected T cell clone (11.65). The horizontal axis indicates the nucleotide position in the full-length HTLV-1 provirus (J02029), and the vertical axis the read depth (arbitrary units). The reads that aligned within the LTRs are indicated in grey. The bars on the horizontal axis indicates the LTRs.
