# Supplementary material for: Epigenetic changes around the pX region and spontaneous HTLV-1 transcription are CTCF-independent
Source: Wellcome Open Res. 2018 Dec 11;3:105. Originally published 2018 Aug 24. [Version 2] doi: 10.12688/wellcomeopenres.14741.2 (PMC6305241; doi:10.12688/wellcomeopenres.14741.2)
Supplement: Supplementary file 2 [file wellcomeopenres-3-16324-s0005.tgz › a38741a2-2c46-485a-b00a-ff0da207890d_revised_Supplementary_Figure_2.pdf]

Spontaneous HTLV-1 transcription and epigenetic changes around the pX region are CTCF-independent.

Miura M *et al*. *Wellcome Open Res* 2018

Supplementary material

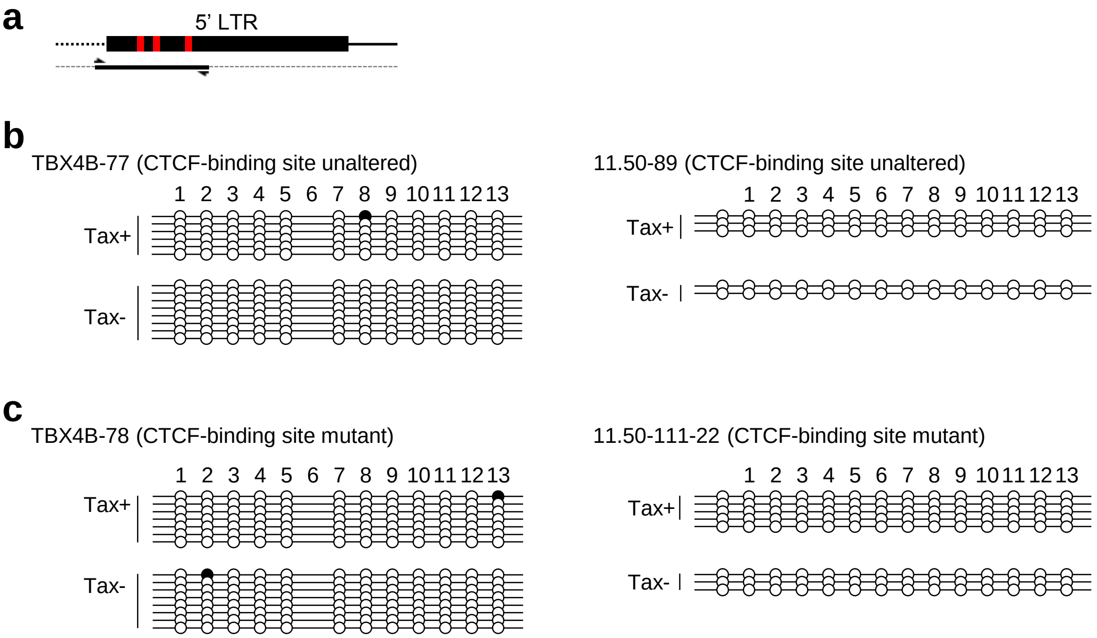

Supplementary Figure 2 DNA methylation in the 5' LTR promoter in HTLV-1-infected clones

(a) Region amplified by PCR for bisulfite sequencing. The three Tax-responsive elements are indicated in red. (b) Schematic representation of DNA methylation. Each bar represents a PCR fragment that was cloned and sequenced. Open circles indicate unmethylated cytosine, and closed circles methylated cytosine. The numbers correspond to the positions shown in Figure 5a. The HTLV-1 T cell clone TBX4B does not have CpG at position 6, so this position is omitted. (c) DNA methylation in the 5' LTR promoter in mutated HTLV-1-infected clones that lack CTCF binding in the provirus.
