# Supplementary material for: Epigenetic changes around the pX region and spontaneous HTLV-1 transcription are CTCF-independent
Source: Wellcome Open Res. 2018 Dec 11;3:105. Originally published 2018 Aug 24. [Version 2] doi: 10.12688/wellcomeopenres.14741.2 (PMC6305241; doi:10.12688/wellcomeopenres.14741.2)
Supplement: Supplementary file 3 [file wellcomeopenres-3-16324-s0006.tgz › 90876f24-e711-4abf-953a-00e9740fde42_revised_Supplementary_Figure_3.pdf]

Spontaneous HTLV-1 transcription and epigenetic changes around the pX region are CTCF-independent.

Miura M *et al*. *Wellcome Open Res* 2018

Supplementary material

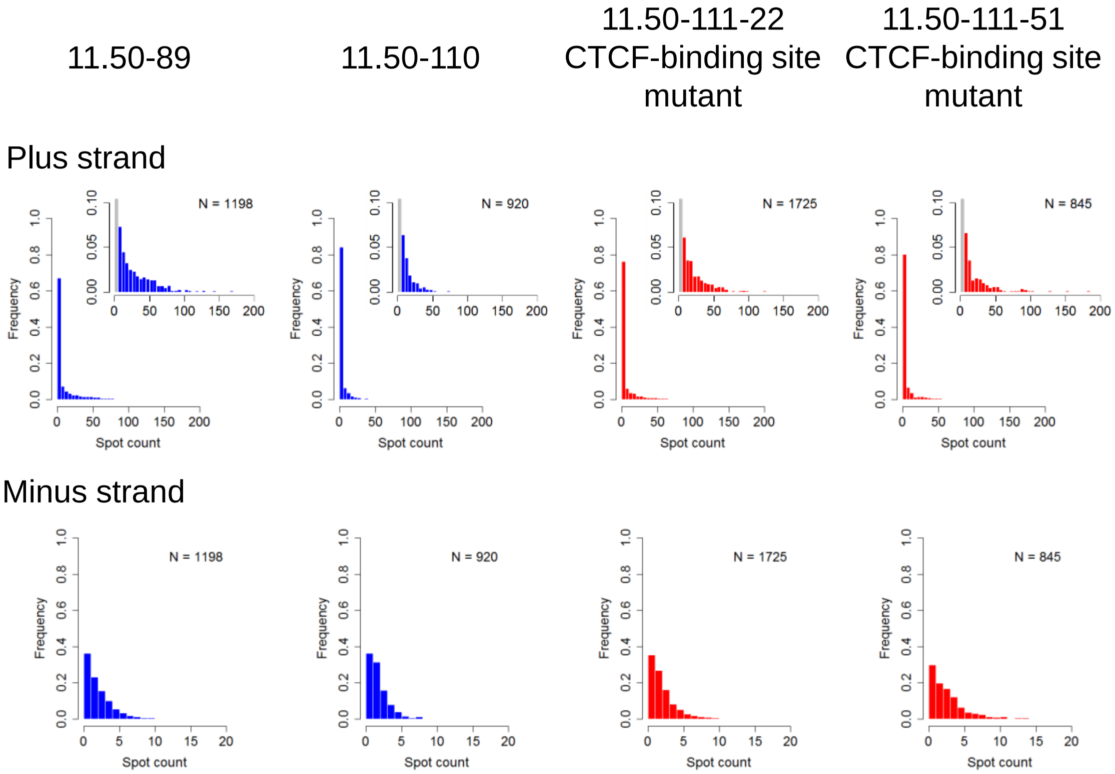

Supplementary Figure 3 Count of viral transcripts in an HTLV-1⁺ T cell clone (Clone 11.50)

Number of spots per cell of the plus-strand (upper row) and minus-strand (bottom row) transcripts in the subclones of 11.50, identified by smFISH. The number of cells analysed is indicated in each panel. The inset in the plus-strand transcription highlights the low-frequency events on a magnified y axis; the bar in the first bin in the insets is greyed out because it is out of scale.
