# Supplementary material for: Epigenetic changes around the pX region and spontaneous HTLV-1 transcription are CTCF-independent
Source: Wellcome Open Res. 2018 Dec 11;3:105. Originally published 2018 Aug 24. [Version 2] doi: 10.12688/wellcomeopenres.14741.2 (PMC6305241; doi:10.12688/wellcomeopenres.14741.2)
Supplement: Supplementary file 4 [file wellcomeopenres-3-16324-s0007.tgz › ab9b6570-8fe9-4bef-ac81-fcb39e435497_Revised_supp_file_1.pdf]

Spontaneous HTLV-1 transcription and epigenetic changes around the pX region are CTCF-independent.

Miura M *et al*. *Wellcome Open Res* 2018

Supplementary File 1

Table 1. PCR primers used for methylated DNA immunoprecipitation and qPCR

Target	Forward	Reverse	Probe [6FAM] - [TAM]
5' LTR	GACAGCCCATCCTATAGCACTC	CTAGCGCTACGGGAAAAGATT	TCGTCCGGGATACGAGCGCC
gag	CAGAGGAAGATGCCCTCCTATT	GTCAACCTGGGCTTTAATTACG	TACCGTTAGATCCCGCCCGTCG
pol	CAGCCCATTCGGCAAG	TGAGAGTAGTAGTAGGTCCTCATGG	CATGGATGACATTCTCCTAGCAAGCCC
pol2	CGTGGCGCTGTCTCAAC	CTGCGAACGTGGTGCAA	TGGGCACCTTCCAAGGCAGGTC
env	GCTAGTTCTGCCCAGTGGAT	TTGGTGGTCTTTTTCTTTGG	CTGCATGCCCAAGACCCGTCG
env2	ACTGGACCCACTGCTTTGAC	ATCCTAGGGTGGGAACAGGT	CCCTGTCATAACTCCCTCATCCTGCC
pX	CCGATCACGATGCGTTTC	ACGGTTTGCTATCCTTAGAAGA	TGGAGGGCCCCGTCGCA
pX2	CTCCTTCCGTTCCACTCAAC	GTGGTAGGCCTTGGTTTGAA	CGCCTATGATTTCCGGGCCCTG
3' LTR	AATACACCAACATCCCCATTTC	GTTTTTCACTGGGAGGCTCTAA	AGAGGCAGATGACAATGACCATGAGCC

Table 2. PCR primers and annealing temperature to amplify bisulfite-treated HTLV-1 provirus

Target	Nested	Forward	Reverse	Annealing
c	1st	YGATGGTAYGTTTATGATTTTYGGG[1]	ACCCCCTCCTAAACTATCTCC[1]	53ºC
	2nd	TTTGTTTGAAGAATATATTAATATT	AACTCCTACTAATTTATTAAACC[1]	48ºC
d	1st	TGATAATGATTATGAGTTTTAAATATTT	ACCCCCTCCTAAACTATCTCC[1]	52ºC
	2nd	TGATAATGATTATGAGTTTTAAATATTT	AACTCCTACTAATTTATTAAACC[1]	48ºC
e	1st	TYGGGAAGTTATYGGAATTATTTATT	TAAAATTAAACAAACAAAATCAAACAA	48ºC
	2nd	TTATGTTTGTTAAGTYGTTTTTAGG	TAAAATTAAACAAACAAAATCAAACAA	48ºC
f	1st	TTTGGAGTTTATTTAGATTTAG[1]	CCAATAATAAACRACCAACCC[1]	45ºC
	2nd	GTTTTGTTTGATTTTGTTTGT[1]	AAAAAAATTTAACCCATTACC[1]	49ºC

Reference

1. Taniguchi, Y. *et al.* Silencing of human T-cell leukemia virus type I gene transcription by epigenetic mechanisms. *Retrovirology* **2**, 64 (2005).
