# Supplementary material for: Epigenetic changes around the pX region and spontaneous HTLV-1 transcription are CTCF-independent
Source: Wellcome Open Res. 2018 Dec 11;3:105. Originally published 2018 Aug 24. [Version 2] doi: 10.12688/wellcomeopenres.14741.2 (PMC6305241; doi:10.12688/wellcomeopenres.14741.2)
Supplement: Peer review history

# Peer review: Referee response for version 2

**DOI:** 10.21956/wellcomeopenres.16324.r34437

**Published:** 2018-12-21

**Authors:** Paul M Lieberman

**Affiliations:** 1 Gene Expression & Regulation Program, The Wistar Institute, Philadelphia, PA, USA

I have read this submission. I believe that I have an appropriate level of expertise to confirm that it is of an acceptable scientific standard.

# Peer review: Referee response for version 1

**DOI:** 10.21956/wellcomeopenres.16060.r33755

**Published:** 2018-09-14

**Authors:** Vincenzo Ciminale

**Affiliations:** 1 Department of Surgery, Oncology and Gastroenterology, University of Padua, Padua, Italy

In the present study Miura et al., carried out a study aimed at investigating the epigenetic changes in the HTLV-1 genome that take place upon (re)activation of viral transcription.

 Based on their findings the authors conclude that the histone modifications H3K4me3, H3K9Ac and H3K27Ac correlated with plus-strand expression. Furthermore, plus-strand transcription was associated to promoter hypomethylation. This pattern of viral epigenetic modifications appeared to be independent of both the provirus insertion site and the binding of the CTCF chromatin architectural protein.

 The paper is clearly written and the pertinent literature is appropriately cited and discussed.

 The experiments are well laid-out and technically sound. The conclusions are well supported by the results and address an important aspect of the HTLV-1 life cycle. The methods are well described and appropriate.

 As the T-cell clones show a mixture of Tax positive and Tax-negative cells, it would be very interesting to synchronize viral expression in these cells (e.g. by cell cycle double block or by reversible inhibition of transcription) to better follow the temporal regulation of these expression bursts. This would also be an ideal model to study the mechanisms controlling such rapid changes in histone acethylation and DNA methylation.

 Minor points.

 Page 3

 The authors describe the effects of Tax on cell turnover. It would be fair to mention its powerful  effect on plus strand transcription.

 The author state that, upon ex vivo culture of primary cells from infected patients, plus-strand expression is greatly increased while, the abundance of HBZ mRNA remains relatively stable. Although there is little doubt about the fact that plus strand expression is increased, previous studies showed that HBZ expression is increased as well (Rende et al., 2011).

 Page 6

 The CHIP and MeDIP analyses were carried out on different patients. It would have been more informative to compare these results in the same patients.

 Page 10

 The term CTCF mutant may be misleading as the mutation was introduced in the CTCF-binding site in the viral genome - not in the CTCF gene.

I have read this submission. I believe that I have an appropriate level of expertise to confirm that it is of an acceptable scientific standard.

# Response

**Published:** 2018-11-30

**Authors:** Charles Bangham

**Affiliations:** Department of Immunology, Imperial College London, UK

**Minor points.**

Page 3

The authors describe the effects of Tax on cell turnover. It would be fair to mention its powerful  effect on plus strand transcription.

Response.  A sentence mentioning the positive feedback by Tax on the 5’ LTR promoter has been inserted in the 3rd paragraph in the Introduction section.

The author state that, upon ex vivo culture of primary cells from infected patients, plus-strand expression is greatly increased while, the abundance of HBZ mRNA remains relatively stable. Although there is little doubt about the fact that plus strand expression is increased, previous studies showed that HBZ expression is increased as well (Rende et al., 2011).

Response.  We are currently investigating the quantity of *HBZ* mRNA in patient-derived PBMCs by smFISH. Therefore, in the present paper we confine the discussion on the HBZ expression in ex vivo culture.

Page 6

The CHIP and MeDIP analyses were carried out on different patients. It would have been more informative to compare these results in the same patients.

Response.  Thank you for pointing out. We agree that it is informative to discuss results of different assays (ex. ChIP and MeDIP) in same patients, and also to compare the difference between patients for a same assay. To enable comparison of the results in this paper with data on the same subjects reported in other publications from our group, we have revealed in the revised paper the three-letter patient codes that are used in our other publications.

Page 10

The term CTCF mutant may be misleading as the mutation was introduced in the CTCF-binding site in the viral genome - not in the CTCF gene.

Response.  We have systematically replaced the term “CTCF mutant” with “ΔCTCF-binding clone”.

# Peer review: Referee response for version 1

**DOI:** 10.21956/wellcomeopenres.16060.r33758

**Published:** 2018-08-31

**Authors:** Paul M Lieberman

**Affiliations:** 1 Gene Expression & Regulation Program, The Wistar Institute, Philadelphia, PA, USA

HTLV-1 has complex transcriptional regulation in early and established long-term infections in T-cells.   The Bangham group has previously shown that HTLV1 genomes establish an epigenetic patterns in latently infected T-cells, and how this corresponds to differences in viral transcription from the positive (Tax+) and negative (HBZ+) strands.  Several intriguing features were previously observed, including a boundary of DNA methylation and a CTCF binding site at the pX locus.  Additionally, a single cell study showed that transcription from the positive strand was dynamic, with some rare clonal bursts of TAX expression and viral reactivation, while the negative stand expression of HBZ was constitutive in all populations.  In this new study, the authors further characterize the epigenetic control of HTLV-1 infected PBMCs and T-cell clones by comparing populations actively expressing Tax from those not expressing Tax.

 The authors show by ChIP-Seq that histone modifications associated with transcription are activation at the 5’ LTR and genome body of the Tax expressing population of HTLV-1 cells.  In contrast, DNA methylation patterns as measured by MeDIP do not show significant variation in T-cell clones whether or not they express Tax.  Sequence specific DNA methylation as measured by bisulfite sequencing shows some differences in Tax positive relative to Tax negative PBMCs from HTLV-1 carriers.  Another major conclusion is that the CTCF binding to the proviral genome has no measurable function on viral gene expression or epigenetic patterning.  Although this CTCF mutation is essentially negative data, it is important to report in context to previously observed findings and expectations of CTCF function in regulation viral transcription and epigenetics.

 Specific Comments

 1.   The dynamic expression of the HTLV-1 positive strand needs some further characterization. What percentage of cells express Tax in the PBMC carriers  and in the T-cell clones? Is this cell cycle, replicative age, or stress dependent? 

 2.  The evidence that the population not expressing TAX is still expressing the negative strand is the single cell FISH.  Can this also be detected by strand-specific RT-PCR to validate the FISH?

 3.  Fig. 3.  ChIP-Seq data excludes the LTR.  Understandably, since the LTR is duplicated, it may be problematic to assign specific signals to the 5’ or 3’ region.  Nevertheless, the analysis of the combined LTR could be informative and should be included in the manuscript, either as a separate panel, or for a representative 5’ or 3’ LTR region for each histogram 

 4.  Fig 3B does not provide a ChIP-Seq for CTCF on PBMCs.  This is problematic since the paper focuses on the role of CTCF in various infection and latency scenarios.   If CTCF does not bind in PBMCs, this should be reported, and the temporal or stochastic nature of CTCF binding should be further investigated.  Is CTCF binding dependent on culture condition or clone specific?

 5.  Fig 9 is difficult to interpret without additional controls.  A positive and negative control for CTCF binding, as well as an IgG control,  should be included in each sample (Tax+ vs Tax-).  Without these controls, or without the CTCF ChIP-Seq data in PBMCs from fig 3B, it is not possible to conclude that CTCF binds to the majority of genomes during primary infection in PBMCs.  This raises the issue of whether CTCF binding to the HTLV1 genome is highly variable for different viral integrations and isolated clones.  Along these lines, it may be worth examining multiple clonal isolates to determine how consistently CTCF binds to the proviral genome.

 6.  The title may be misleading since it is not clear that the 5’ and 3’ LTRs are analyzed completely and separately from each other.

 Minor Issues:

 1.  A reference should be provided  for statement on p3. 3 ^rd^ paragraph of Introduction:  “… transcription of… the plus and minus strand, respectively.  (REF)”

 2.  p. 6, 1 ^st^ column, Results section:  “…In the 3’ UTR and pX region, these three histone marks (H3K4me3, H3K9Ac and H3K27Ac) were constantly detected, regardless of Tax expression.”

 I think this could be stated more clearly.  Perhaps starting with a  statement referring to the Tax negative population.  Also, the 3’UTR is not examined, but rather the boundary adjacent to the 3’ UTR is measured, according to the Figure Legend.    Finally, the levels of H3K4me3 at the 3’ end of the genome appears to be significantly enriched relative to the Tax positive population, perhaps suggesting these cells are expressing negative strand RNA and HBZ.  This could be tested experimentally.

 3.  p6, 2 ^nd^ column, 1 ^st^ sentence:  “…regardless of viral reactivation.”    I think “reactivation” may not be the appropriate designation, as TAX expression is the only readout, and this expression is dynamic through an unknown mechanism that may be distinct from viral reactivation that produces infectious particles.  The status of HBZ expression should be tested or discussed.

 4.  It is surprising that there is so little DNA methylation detected in the LTR of the stable T-cell clones as measured by bisulfite sequencing (Fig. S2) compared to PBMCs from carriers (Fig. 5).   Some consideration of this difference could be included in the Discussion section.

 5.  Fig. 5D.   The bisulfite methylation may suggest that there is a trend toward selective methylation of primer region D in the Tax negative population. If the bisulfite conversion is not complete, this trend may suggest that DNA methylation is the mechanism of epigenetic silencing at the 5’ UTR.

I have read this submission. I believe that I have an appropriate level of expertise to confirm that it is of an acceptable scientific standard, however I have significant reservations, as outlined above.

# Response

**Published:** 2018-11-30

**Authors:** Charles Bangham

**Affiliations:** Department of Immunology, Imperial College London, UK

**Specific Comments**

 1.   The dynamic expression of the HTLV-1 positive strand needs some further characterization. What percentage of cells express Tax in the PBMC carriers  and in the T-cell clones? Is this cell cycle, replicative age, or stress dependent?

 Response.  In HTLV-1-infected T cell clones, some 5-10% of the total population carry >4 plus-strand transcripts at any one time. We reported in our previous publication (Billman et al. Wellcome Open Research, 2017) that (1) HTLV-1-infected T cell clones have fewer *HBZ* mRNA molecules at the G0/G1 stage, and (2) a plus-strand burst (i.e. a rapid transcription) is more likely to be observed when a cell lacks detectable *HBZ* mRNAs. Indeed, the odds of a cell having a plus-strand burst at the G2/M stage is significantly lower than in cells in G0/G1 (Billman et al. 2017). These observations indicate an association between the plus-strand expression and the cell cycle in HTLV-1 T cell clones cultured in vitro.

 In PBMCs isolated from HTLV-1 carriers, the HTLV-1 plus-strand reactivation is likely to be caused by environmental stress, as is observed in many other viruses. We are currently investigating the frequency and intensity of the HTLV-1 plus- and minus-strand expression during ex vivo culture of PBMCs by smFISH.

 Paragraph 5 in the Introduction has been modified to include these important points, and the appropriate papers cited.

 2.  The evidence that the population not expressing TAX is still expressing the negative strand is the single cell FISH.  Can this also be detected by strand-specific RT-PCR to validate the FISH?

 Response.  A possible alternative approach would be to sort the cells by flow cytometry for Tax protein and assay HBZ (minus strand) in the Tax-positive and Tax-negative fractions by RT-PCR. However, such approaches have certain limitations. Tax protein detection is less sensitive than smFISH, and crucially there is a time-lag between the onset of *tax* mRNA expression and protein expression; third, the PCR does not give information at the single-cell level.  At present, we believe that smFISH is the most sensitive and specific technique available to detect mRNA, especially at the single-cell level.

 3.  Fig. 3.  ChIP-Seq data excludes the LTR.  Understandably, since the LTR is duplicated, it may be problematic to assign specific signals to the 5’ or 3’ region.  Nevertheless, the analysis of the combined LTR could be informative and should be included in the manuscript, either as a separate panel, or for a representative 5’ or 3’ LTR region for each histogram

 Response.  We have revised Figure 3, Figure 7a and Supplementary Figure 1 to include reads aligned within the LTRs. We present those reads with grey colour in such a way that they can be differentiated from the rest of the reads that uniquely aligned.

 4.  Fig 3B does not provide a ChIP-Seq for CTCF on PBMCs.  This is problematic since the paper focuses on the role of CTCF in various infection and latency scenarios.   If CTCF does not bind in PBMCs, this should be reported, and the temporal or stochastic nature of CTCF binding should be further investigated.  Is CTCF binding dependent on culture condition or clone specific?

 Response.  In Figure 3b, we omitted the ChIP-seq for CTCF in patient-derived PBMCs because of the limited amount of materials. In the revised Figure 9 we show that HTLV-1 binds CTCF in PBMCs regardless of the plus-strand expression. Also, in our previous publication reporting the binding of CTCF in the HTLV-1 provirus (Satou et al. PNAS, 2016), we demonstrated that CTCF binds to various HTLV-1-infected cell lines as well as PBMCs isolated from HTLV-1-infected individuals.

 It is documented that CTCF-binding is affected by methylation at one of the cytosine residue in the CTCF binding-site. In HTLV-1, CTCF binds to the pX region in the provirus where DNA is rarely methylated (Taniguchi et al 2005, op.cit.) (Figure 4). Therefore, we anticipate that CTCF binding to the HTLV-1 provirus shows little variation among culture conditions or provirus insertion sites. However, it should be noted that a lack of variation in CTCF-binding to HTLV-1 does not necessarily mean that the CTCF binding is strong. We discuss the CTCF occupancy as a surrogate measure of binding strength in the next point.

 5.  Fig 9 is difficult to interpret without additional controls.  A positive and negative control for CTCF binding, as well as an IgG control,  should be included in each sample (Tax+ vs Tax-).  Without these controls, or without the CTCF ChIP-Seq data in PBMCs from fig 3B, it is not possible to conclude that CTCF binds to the majority of genomes during primary infection in PBMCs.  This raises the issue of whether CTCF binding to the HTLV1 genome is highly variable for different viral integrations and isolated clones.  Along these lines, it may be worth examining multiple clonal isolates to determine how consistently CTCF binds to the proviral genome.

 Response.  In the revised paper, we show the results (revised Figure 9) of new experiments in which we carried out CTCF ChIP assay on patient-derived PBMCs with two biological replicates, including a negative control (rabbit IgG) and the TC-1 locus as a positive control.  The results show that CTCF binds to HTLV-1 after overnight culture, regardless of plus-strand expression. This observation is consistent with the conclusion that CTCF-binding has no discernible immediate impact on HTLV-1 expression.

 The results in the revised Figure 9 also show that the CTCF ChIP signal at the HTLV-1 CTCF-binding site was lower than at the TC-1 locus (positive control for CTCF binding).  CTCF occupancy (an approximation of binding strength) depends on the primary DNA sequence (Liu et al. Nature Biotechnology, 2015) and   can be estimated by ChIP readout.  We infer that CTCF occupancy at the HTLV-1 provirus is lower than that at TC-1.

 6.  The title may be misleading since it is not clear that the 5’ and 3’ LTRs are analyzed completely and separately from each other.

 Response.  We acknowledge that we have not explicitly identified the epigenetic modifications within the two LTRs. Also, as pointed out by both of the reviewers, an important finding is that CTCF appeared to have no discernible impact on the epigenetic modifications around the pX region or on spontaneous transcription. Therefore, we have altered the title of the revised paper: Spontaneous HTLV-1 transcription and epigenetic changes around the pX region are CTCF-independent.

**Minor Issues:**

 1.  A reference should be provided  for statement on p3. 3rd paragraph of Introduction:  “… transcription of… the plus and minus strand, respectively.  (REF)”

 Response.  A reference (Kulkarni et al. 2018, review) has been inserted (Ref #5 in the revised paper).

 2.  p. 6, 1st column, Results section:  “…In the 3’ UTR and pX region, these three histone marks (H3K4me3, H3K9Ac and H3K27Ac) were constantly detected, regardless of Tax expression.”

 I think this could be stated more clearly.  Perhaps starting with a  statement referring to the Tax negative population.  Also, the 3’UTR is not examined, but rather the boundary adjacent to the 3’ UTR is measured, according to the Figure Legend.    Finally, the levels of H3K4me3 at the 3’ end of the genome appears to be significantly enriched relative to the Tax positive population, perhaps suggesting these cells are expressing negative strand RNA and HBZ.  This could be tested experimentally.

 Response.  Thank you for the suggestion. In the revised paper, we have defined the terms “5’ LTR junction” and “3’ LTR junction” at the beginning of the Results section, to clarify how we refer to the regions adjoining the 5’ LTR and 3’ LTR.  Subsequently we systematically replaced “5’ LTR” and “3’ LTR” with “5’ LTR junction” and “3’ LTR junction” respectively.

 In relation to this comment, two sentences have been altered in the revised paper: (1) In the last sentence in the first paragraph (Page 11) (“These results are inconsistent with the idea that CTCF imposes an epigenetic border in the HTLV-1 provirus and regulates the distinct modifications between the 5′ and 3′ LTRs.”), we replaced the phrase “between the 5’ and 3’ LTR” with “around the pX region”. (2) In the last sentence in the Discussion section, we replaced “in the 5’ and 3’ LTRs in HTLV-1” with “around the pX region in HTLV-1”.

 Lastly, we state in the revised paper that H3K4me3 at the pX and adjoining the 3’ LTR is higher in the Tax– population, presenting additional evidence obtained by ChIP-qPCR to make a quantitative comparison. The new data are presented in the revised Supplementary Figure 1 (Supplementary Figure 1a).

 3.  p6, 2nd column, 1st sentence:  “…regardless of viral reactivation.”    I think “reactivation” may not be the appropriate designation, as TAX expression is the only readout, and this expression is dynamic through an unknown mechanism that may be distinct from viral reactivation that produces infectious particles.  The status of HBZ expression should be tested or discussed.

 Response.  We have preliminary evidence from smFISH analysis of HTLV-1 ^+^ PBMCs that cells that restore the plus-strand transcription also produce unspliced transcripts ( *gag*), which is associated with production of HTLV-1 infectious particles. HBZ expression is currently under investigation with smFISH in conjunction with plus-strand transcripts at the single-cell level.

 4.  It is surprising that there is so little DNA methylation detected in the LTR of the stable T-cell clones as measured by bisulfite sequencing (Fig. S2) compared to PBMCs from carriers (Fig. 5).   Some consideration of this difference could be included in the Discussion section.

 Response.  There are two major points to discuss concerning the difference between HTLV-1 ^+^ T cell clones and PBMCs. First, single T-cell clones are isogenic in terms of the genomic environment of the provirus insertion site, whereas the polyclonal population of PBMCs contains proviruses inserted in tens of thousands of unique sites, each with a distinct genomic environment. Second, it is plausible that a single cell with a particular pattern of DNA methylation was selected at the beginning of T cell cloning, and that pattern has been stably inherited in the clone.  We infer that, at least in in vitro culture, DNA methylation at the 5’ LTR promoter is stable, and has little impact on the spontaneous expression of the plus strand. We have added this point in the paragraph entitled “DNA hypomethylation in the plus-strand promoter is a prerequisite for viral reactivation” in the Results section.

 5.  Fig. 5D.   The bisulfite methylation may suggest that there is a trend toward selective methylation of primer region D in the Tax negative population. If the bisulfite conversion is not complete, this trend may suggest that DNA methylation is the mechanism of epigenetic silencing at the 5’ UTR.

 Response.  Our results show that the bisulfite conversion was highly efficient, as we could hardly see cytosine residues at non CpG sites after conversion.  To estimate to what degree DNA methylation accounts for the provirus silencing, independently of the position effect, would require simultaneous identification of insertion site, DNA methylation and virus transcription in single cells. However, this is beyond the scope of the present paper.
